# Systematic review with network meta-analysis of antivascular endothelial growth factor use in managing polypoidal choroidal vasculopathy

**DOI:** 10.1038/s41598-021-82316-y

**Published:** 2021-02-02

**Authors:** Sheng-Chu Chi, Yi-No Kang, Yi-Ming Huang

**Affiliations:** 1 Department of Ophthalmology, Taiwan Faculty of Medicine, Taipei Veterans General Hospital, Taipei, Taiwan; 2 Evidence-Based Medicine Center, Wan Fang Hospital, Taipei Medical University, Taipei, Taiwan; 3 Research Center of Big Data and Meta-Analysis, Wan Fang Hospital, Taipei Medical University, Taipei, Taiwan; 4 Cochrane Taiwan, Taipei Medical University, Taipei, Taiwan; 5 Institute of Health Policy and Management, College of Public Health, National Taiwan University, Taipei, Taiwan; 6 National Yang-Ming University School of Medicine, Taipei, Taiwan

**Keywords:** Retinal diseases, Vision disorders

## Abstract

Polypoidal choroidal vasculopathy (PCV) is a vision-threatening disease common in Asian populations. However, the optimal treatment for PCV remains under debate. We searched the databases with optimal searching strategy. The study included randomized clinical trials and prospective studies that recruited patients with active PCV who had received interventions, including PDT, anti-VEGF, or a combination of PDT and anti-VEGF. The Grading of Recommendations Assessment, Development, and Evaluation methodology was used for rating the quality of evidence. Our study included 11 studies involving 1277 patients. The network meta-analysis of RCTs revealed the anti-VEGF group, early combination group, and late combination group had significant BCVA changes compared with the PDT group. Early combination therapy led to a significant decrease in CRT compared with PDT, anti-VEGF, and late combination therapy. Additionally, the early combination group had a significantly higher complete polyp regression rate than the anti-VEGF group. No significant differences were detected in the analysis of the number of anti-VEGF injections and safety profile. This network meta-analysis revealed that early combination therapy exhibited better efficacy related to anatomical outcomes than other therapies. Nonetheless, no significant differences related to BCVA change could be detected between anti-VEGF and late combination therapy.

## Introduction

Polypoidal choroidal vasculopathy (PCV) is considered a vision-threatening retinal disease. It is characterized by an abnormal inner choroidal branching vascular network with a nodular polypoidal aneurysmal lesion. The clinical features of PCV include reddish-orange nodular structures beneath the retina, serous pigment epithelial detachment, retinal pigment atrophy, and serous neurosensory detachment^1^. Antivascular endothelial growth factor (anti-VEGF) agents have been the first-line therapy for PCV because of their efficacy and safety^2,3^. Moreover, anti-VEGF can be combined with photodynamic therapy (PDT) for treating PCV^4^. PDT is another common treatment for PCV that targets the endothelial cells of vessels, resulting in the selective occlusion of polyp vessels and the resolution of active macular edema^5^. Notably, PDT can be used alone or in combination with anti-VEGF to treat PCV^1,6^. However, choosing the appropriate treatment for PCV is still a critical aspect of clinical practice.

Several prospective observational studies and randomized clinical trials (RCTs) have reported the efficacy of anti-VEGF agents, PDT, and their combination for treating PCV^7–18^. These studies have further subdivided combined therapy into early combination and late combination therapy. Notably, in early combination therapy, a patient receives PDT and anti-VEGF therapy at the beginning of the treatment course. However, in late combination therapy, patients receive anti-VEGF first, followed by rescue PDT.

Synthesized evidence from 4 studies was published before 2019^19–22^, and the latest meta-analysis derived conclusions from heterogeneous findings due to mixed data from retrospective studies and 2 RCTs. Notably, conceptual heterogeneity is unavoidable Consequently, the appropriate treatment strategy for PCV remains controversial.

Furthermore, several new RCTs were completed and published in 2017 and 2018^10,13,14^. Therefore, an updated synthesis might confirm existing evidence and provide insight into PCV treatment. Hence, we conducted a systematic review with network meta-analysis to compare the effects of PDT monotherapy, anti-VEGF monotherapy, early combination therapy, and late combination therapy in treating PCV in terms of BCVA improvement, anatomical changes, and safety.

## Methods

This systematic review with network meta-analysis of prospective studies was conducted to better understand the efficacy of PDT, anti-VEGF, and combination therapies in treating PCV; the study was designed in accordance with the Cochrane Handbook for Systematic Reviews of Interventions and registered on PROSPERO (CRD:42,020,181,736) beforehand. This synthesis was performed in accordance with the Preferred Reporting Items for Systematic Reviews and Meta-Analyses guidelines^9^. Institutional review board approval was not deemed necessary because the study used published data for analysis.

### Eligibility criteria and evidence selection

Predefined eligibility criteria for evidence selection were as follows: (1) RCT or prospective study design; (2) study of patients with active PCV; and (3) use of interventions with PDT, anti-VEGF, or a combination of PDT and anti-VEGF. Exclusion criteria were as follows: (1) studies with unreported outcomes, for BCVA, proportion of patients with complete polyp regression, central retinal thickness (CRT) decrease, rates of adverse or ocular adverse events; (2) gray literature lacking a detailed report. On the basis of the different treatment modalities in the included trials, combination therapy was further subdivided into early combination and late combination therapy. Patients assigned to the late combination group where those who did not receive PDT initially but later received rescue PDT. Potential references were identified from the Cochrane Library, EMBASE, and New PubMed before February 2020. The search strategy consisted of using relevant terms such as “PCV,” “PDT,” and “anti-VEGF” in the free text and medical subject heading and using Boolean algebra. Details are provided in the Supplementary 1. Two reviewers (SCC and YNK) independently reviewed the references identified from the databases. Duplicates and irrelevant references were excluded through the screening of titles and abstracts after the search, and we retrieved full texts for further review of the remaining articles.

### Data extraction and quality assessment

The 2 reviewers (SCC and YNK) independently identified and extracted relevant information, including study year; population characteristics; treatment modality; authors of studies; and outcomes, such as BCVA change, BCVA improvement rate, CRT decrease, the proportion of patients with complete polyp regression, number of anti-VEGF injections, adverse events, and ocular adverse events. The authors avoided double-count data from same trial or population by double checking relevant information of each trial. We used the Early Treatment Diabetic Retinopathy Study Visual Acuity Chart as the scale for BCVA. For continuous outcomes, mean and SD were extracted. If SE was presented in original trials, then SD was estimated from the sample size according to the relevant formula (SE = SD/√N). If relevant information of SD or SE could not be extracted from the original report, then authors were contacted. Imputation was employed using the maximum SD among eligible trials in the same outcome when the original SD could not be accessed. For instance, for the outcome of CRT decrease, Lee et al. and Koh et al. (2017) did not report relevant information for SD^10,14^, and imputation was performed. For binary outcomes, we extracted the event and total sample sizes.

With reference to relevant information regarding patient characteristics and outcomes, the 2 reviewers (SCC and YNK) independently assessed the bias of the included studies in the network meta-analysis by using the Risk of Bias 2 for RCTs and the Risk Of Bias in Non-randomized Studies of Interventions assessment. In addition, the Grading of Recommendations Assessment, Development, and Evaluation (GRADE) methodology was employed to rate the quality of evidence.

### Data synthesis and analysis

This synthesis involved qualitative and quantitative analyses. All eligible studies were included in the qualitative synthesis, but only data from eligible RCTs were used in network meta-analysis to ensure high-quality evidence synthesis. All analyses were conducted using the random-effects model due to clinical heterogeneity. We reported continuous outcomes, in terms of weighted mean difference (WMD) and 95% CI. Dichotomous outcomes were reported using the risk ratio (RR) and 95% CI. If an effect size raised clinical concerns without statistical significance, the surface under the cumulative ranking curve (SUCRA) was further determined to demonstrate the effects through hierarchical ranking of interventions.

Inconsistency and small-study effects were analyzed to evaluate the quality of network meta-analysis. Inconsistency analysis involved a loop inconsistency test according to Lu-Ades’ method and the design-by-treatment interaction model. The primary method applied in the analysis of this synthesis was loop inconsistency, but the design-by-treatment interaction model was employed when an outcome was contributed by various arm designs. The adjusted funnel plot with Egger’s test was employed for testing small-study effects. We assessed the statistical heterogeneity by using the I^2^ statistic.All analyses were performed using STATA version 14.

## Results

Overall, 1036 potential articles were identified from the Cochrane Library (k = 172), EMBASE (k = 381), and New PubMed (k = 483). After the removal of duplicates through systematic (EndNote) and manual matching, the remaining 747 articles were assessed. Finally, 4 prospective cohort studies^12,15,16,18^ and 7 RCTs^7–11,14^ were included. The flow diagram is illustrated in Supplementary 2.

### Characteristics and quality of included studies

**Figure 1:**
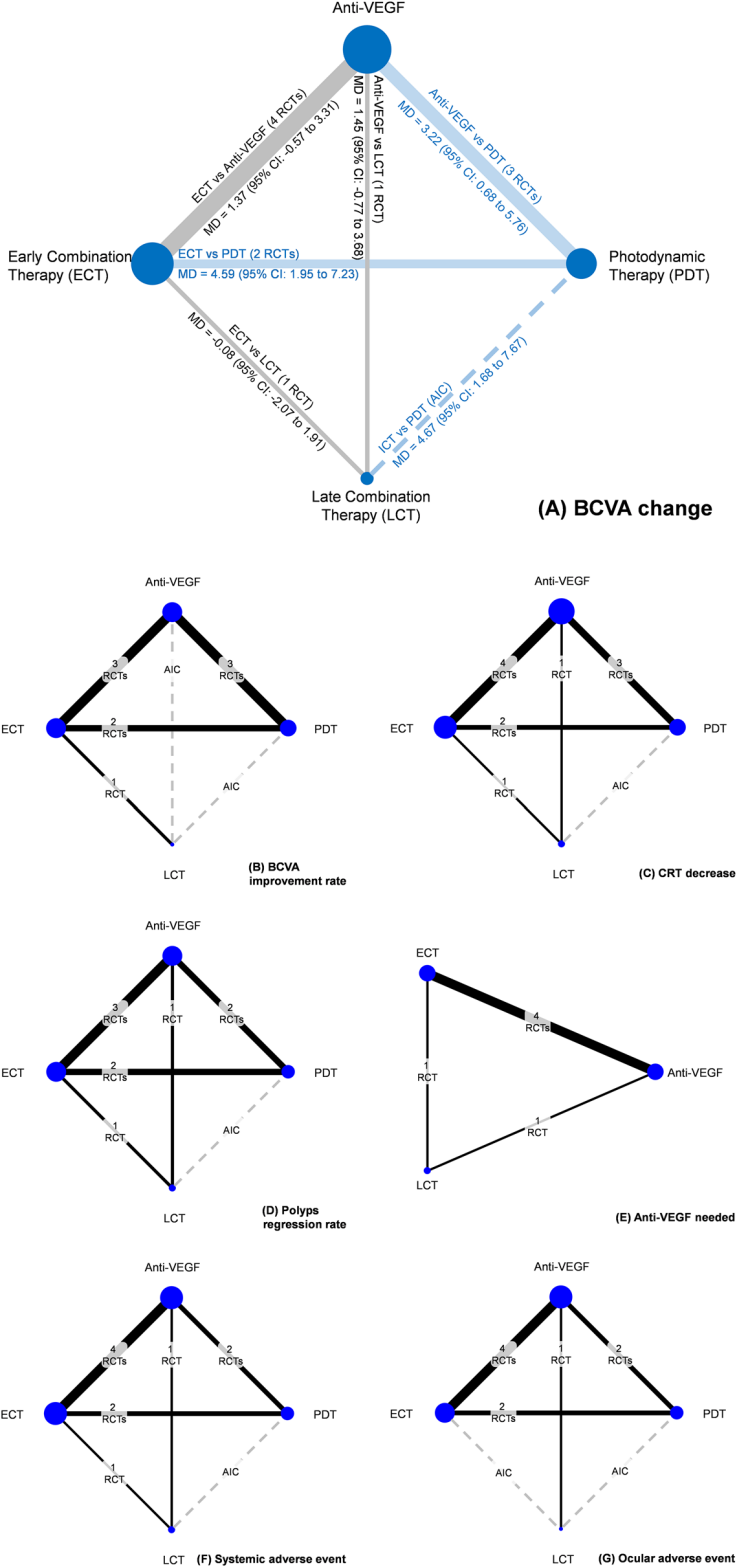
Network plots.

**Figure 2:**
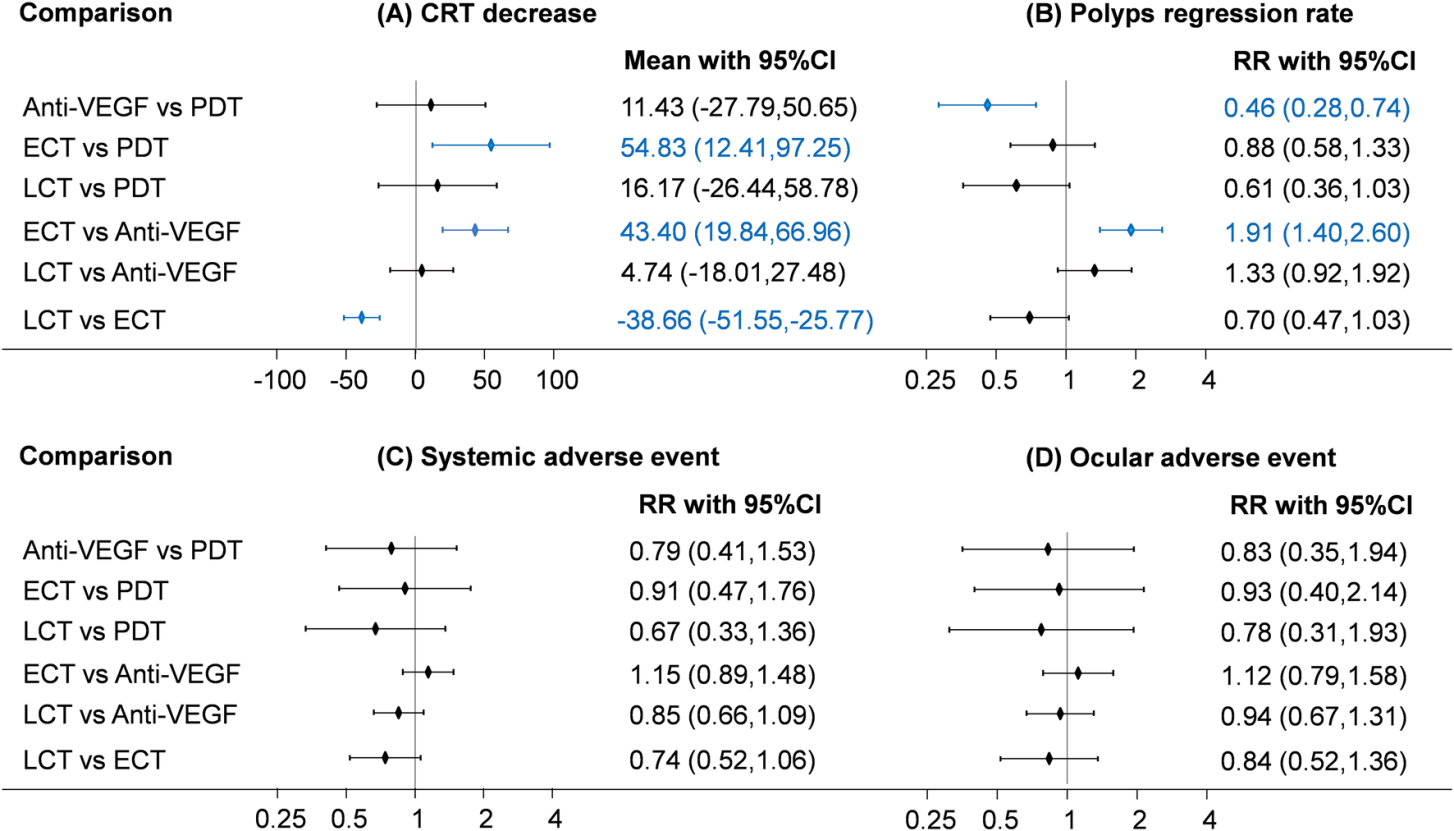
Forest plot of main findings.

Overall, 1277 patients were involved in the 11 studies (7 RCTs and 4 prospective cohort studies). Overall, there were 812 men. The available information revealed that the mean age of participants ranged from 61.98 to 73.70 years, and the baseline BCVA ranged from 38.5 to 63 letters. Some studies employed the central subfield thickness^10,14^ or central foveal thickness^13^ as scales to report the CRT. The baseline CRT among these participants ranged from 254.50 to 506.18um. Details of the characteristics of the included studies are summarized in Table 1. The anti-VEGF agents, PDT protocol, rescue therapy, and criteria of rescue therapy are listed in Table 2. Most of the trials used ranibizumab as the anti-VEGF agent. Regarding PDT protocol, most trials employed standard-fluence PDT. The results of the study appraisal are summarized in Supplementary 3– 4. The network plot of primary outcome was illustrated in Fig. 1, and forest plots of other main finding was presented ion Fig. 2. Summary of other outcomes was showed in Table 3. No asymmetry was noted upon visual inspection of all funnel plots (Supplementary 5–11) No inconsistency or small-study bias was noted in analysis of all outcomes. (Supplementary 12–18) Furthermore, SUCRA value was demonstrated in BCVA change, BCVA improvement rate and number of anti-VEGF needed. (Supplementary 19–21).

**Table 1:** Characteristics of studies.

Study	Study type	Year	Follow-up time (month)	Treatments	Number (eye)	Age (mean)	Sex (M/F)	BCVA (mean)	CRT (mean)	BCVAchange	CRTdecrease	CompletepolypRegression	Adverseevents
Chen et al	prospective cohort	2018	12	Anti-VEGF Combined	64 whole study	68.7 whole study	41/23 whole study	50 whole study	310.8 whole study	Combined superior to Anti-VEGF	N/A	N/A	N/A
Chong et al	prospective cohort	2018	12	ECT LCT	41 152	70.7 69	18/41 69/112	46 60	N/A	-	N/A	N/A	N/A
Gomi et al	RCT	2015	12	ECT LCT	37 35	73.6 73.8	37/0 35/0	54.3 54.9	360.5 345.6	-	-	-	-
Koh et al	RCT	2012	6	PDT Anti-VEGF ECT	21 21 19	62.2 69.3 63.8	15/6 15/6 11/8	57.2 49.0 56.6	285.3 268.5 334.7	-	ECT superior to Anti-VEGF	PDT, ECT superior to Anti-VEGF	-
Koh et al	RCT	2017	24	Anti-VEGF ECT	154 168	68.2 68	116/38 109/59	61.2 61.1	410.4 415.9 (CSFT)	ECT superior to Anti-VEGF	ECT superior to Anti-VEGF	ECT superior to Anti-VEGF	-
Lai et al	RCT	2018	12	PDT Anti-VEGF ECT	23 18 19	60.52 64.67 61.06	14/9 12/6 10/6	40 32 34	478.04 527.50 522.63 (CFT)	-	-	PDT superior to Anti-VEGF	-
Lee et al	RCT	2018	13	ECT LCT	161 157	70.4 70.8	112/49 110/47	59.0 57.7	346.1 347.8	-	-	-	-
Li et al	prospective cohort	2018	12	Anti- VEGF ECT	16 48	66.12 68.44	9/7 28/20	51.5 50.05	456.58 467.64	ECT superior to Anti-VEGF	ECT superior to Anti-VEGF	-	N/A
Lim et al	RCT	2012	12	Anti- VEGF ECT	5 5	68.6 57.8	5/0 3/2	57.01 50.99	295.6 213.4	-	-	N/A	-
Oishi et al	RCT	2013	12	PDT Anti- VEGF	47 46	75.0 75.4	32/15 28/18	56.5 61	366.8 418.9	Anti-VEGF superior to PDT		N/A	N/A-
Teo et al	prospective cohort	2018	3	Anti- VEGF ECT	13 10	68.7 70.3	7/6 6/4	67 60	352 435.4	-	-	ECT superior to Anti-VEGF	N/A-

-, No significant difference between two comparison; Anti-VEGF, anti-vascular endothelial growth factor; BCVA, best-corrected visual acuity; CRT, central retinal thickness; ECT, early combination therapy; LCT, late combination therapy; PDT, photodynamic therapy; N/A, no information of comparison in the study.

**Table 2:** Treatment strategy.

Study		Anti-VEGF treatment	PDT protocol		Rescue therapy		Rescue criteria	
Chen et al		Ranbizumab 0.5 mg according to local practice in Taiwan	N/A		1. Anti-VEGF group: PRN additional anti-VEGF according to local practice in Taiwan 2. Combined group: PRN additional anti-VEGF and PRN PDT according to local practice in Taiwan		According to local practice in Taiwan	
Chong et al		Monthly bevacizumab, ranbizumab , or aflibercept According to real world practice	N/A		1. Early combination group: PRN additional anti-VEGF + PRN additional PDT 2. Late combination group: PRN additional Anti-VEGF, and allowed PRN PDT (postponed PDT PRN)		According to real world practice	
Gomi et al		Monthly ranbizumab 0.5 mg × 3	Standard fluence 6 mg/m2 689-nm wavelengths 600 mW/cm2 irradiance, 83 s		1. Early combination group: PRN additional anti-VEGF monthly and PRN additional PDT every 3 month 2. Late combination group: additional anti-VEGF monthly and allowed PRN PDT every 3 month (postponed PDT PRN)		1. Anti-VEGF: Decrease BCVA ETDRs letter > 5 2. PDT: BCVA < = 0.7 and polypoidal lesions were seen with subretinal fluid on the ICGA images	
Koh et al. (2012)		Monthly ranbizumab 0.5 mgx3	Standard fluence 6 mg/m2 689-nm wavelengths 600 mW/cm2 irradiance, 83 s		1. PDT group: PRN additional PDT monthly 2. Anti-VEGF group: PRN additional anti-VEGF monthly 3. Early combination group: PRN addition PDT monthly and PRN additional anti-VEGF monthly		mainly driven by ICGA-assessed polyp regression, considering in addition FA leakage and BCVA	
Koh et al. (2017)		Monthly ranbizumab 0.5 mgx3	Standard fluence 6 mg/m2 689-nm wavelengths 600 mW/cm2 irradiance, 83 s		1. Anti-VEGF group: PRN additional anti-VEGF monthly 2. Early combination group: PRN additional anti-VEGF monthly and additional PDT every 3 month		1. Anti-VEGF: Decrease BCVA or presence of OCT anomaly 2. PDT: presence of active PCV (polyps or leakage) on ICGA or FA	
Lai et al		Ranbizumab 0.5 mgx1	Standard fluence 6 mg/m2 689-nm wavelengths 600 mW/cm2 irradiance, 83 s		1. PDT group: PRN additional PDT every 3 month 2. Anti-VEGF group: PRN additional anti-VEGF monthly 3. Early combination group: PRN additional anti-VEGF monthly and additional PDT every 3 month		According to PrONTO study Decrease BCVA ETDRs letter > 5 or CFT increase > 100um in OCT orPED enlargement or macula hemorrhage or new PCV orpersistent fluid on OCT	
Lee et al		Monthly aflibercept 2 mg × 3 then 2-monthly in patient no need rescue therapy)	According to the current Visudyne package labeling		1. Anti-VEGF group: PRN additional anti-VEGF monthly 2. Late combination: PRN additional anti-VEGF monthly and allowed PRN PDT every 3 month ( postponed PDT PRN)		BCVA < 73 letter and (BCVA gain < 5 ETDRS letter or > = 5 but < = 10 ETDRS and PDT might be beneficial) and New or persistent fluid in OCT and presence of active PCV on ICGA	
Li et al		Ranbizumab 0.5mlx3	Reduced fluence 10.5 mg, spot size 800 mm ,Lesion was irradiated for 70″ at 600 mW/cm2 and 42 J/cm2		1. Anti-VEGF group: PRN additional anti-VEGF monthly 2. Early combination group: PRN additional anti-VEGF monthly and additional PDT every 3 month		According to PrONTO study Decrease BCVA ETDRs letter > 5 or CFT increase > 100um in OCT orPED enlargement or macula hemorrhage or new PCV or persistent fluid on OCT or active leakage on FAG	
Lim et al.		Bevacizumab 0.05 ml every 6 weeks			1. Anti-VEGF group: PRN additional anti-VEGF at 18,24,32,48 weeks 2. PRN additional anti-VEGF at 18,24,32,48 weeks 3. Early combination group: PRN additional anti-VEGF monthly and additional PDT		CFT increased by more than 100 um New SRF	
Oishi et al		Monthly ranbizumab 0.5 mgx3	Standard fluence 6 mg/m2 689-nm wavelengths 600 mW/cm2 irradiance, 83 s		1. Anti-VEGF group: PRN additional anti-VEGF monthly 2. PDT group: PRN additional PDT every 1.5 month		According to PrONTO study decrease BCVA ETDRs letter > 5 or CFT increase > 100um in OCT orPED enlargement or macula hemorrhage or new PCV or persistent fluid on OCT or active leakage on FAG	
Teo et al.		Bevacizumab16 people Aflibercept 10 people	N/A		1. Anti-VEGF group: PRN additional anti-VEGF monthly 2. Early combinatio group: PRN additional anti-VEGF monthly		if intraretinal or subretinal fluid persisted

**Table 3:** Summary of outcomes.

Comparison		Studies (n)	Effect size		*I*^2^	Inconsistency		Egger test	
Arm 1	Arm 2		WMD	95% CI	(%)	*χ*^2^	*P*	*t*	*P*
BCVA improvement rate %						4.28	0.12	-1.93	0.10
Anti-VEGF	PDT	3	1.26	0.67 to 3.63	0				
ECT	PDT	2	1.49	0.71 to 3.13	0				
LCT	PDT	AIC	1.61	0.55 to 4.68					
ECT	Anti-VEGF	3	1.18	0.64 to 2.16	52				
LCT	Anti-VEGF	AIC	1.27	0.48 to 3.38					
LCT	ECT	1	1.08	0.50 to 2.33					
Extra anti-VEGF needed (Numbers of injections)						3.25	0.07	0.41	0.70
ECT	Anti-VEGF	4	-0.72	-2.07 to 0.63	89.2				
LCT	Anti-VEGF	1	-1.44	-3.61 to 0.72					
LCT	ECT	1	-0.72	-2.02 to 1.48					

AIC, adjusted indirect comparison; Anti-VEGF, anti-vascular endothelial growth factor; BCVA, best-corrected visual acuity; CI, confidence interval; ECT, early combination therapy; LCT, late combination therapy; PDT, photodynamic therapy; WMD, weighted mean difference.

### Efficacy

#### BCVA change

Regarding the outcome of BCVA change, a total of 7 RCTs revealed this outcome^7–10,13,14,17^. In the consistency model, the results revealed significant differences in BCVA change between the anti-VEGF group and PDT group (WMD: 3.22; 95% CI: 0.68, 5.76), the early combination group and PDT group (WMD: 4.59; 95% CI: 1.95, 7.22), and the late combination group and PDT group (WMD: 4.67; 95% CI: 1.68, 7.67). No significant differences were detected among anti-VEGF, early combination, and late combination groups. Among the prospective cohort studies, all 4 studies reported the data of BCVA change. Teo et al. and Chong reported results similar to our analysis. By contrast, Chen et al. and Li et al. reported that combination therapy led to significantly greater BCVA change than did anti-VEGF monotherapy.

#### BCVA improvement rate

Most trials defined BCVA improvement as an EDTR gain of at least > 15 words. Only Lai et al. set the EDTR gain at this threshold. Of the 7 RCTs, 5 reported this outcome. No significant differences were detected in this network meta-analysis. The SUCRA value revealed that late combination therapy could be the optimal therapy in terms of BCVA improvement (SUCRA value 69.4). Notably, only one non-RCT reported this outcome. Li et al. suggested that the early combination group had a higher BCVA improvement rate than the anti-VEGF group.

### Complete polyp regression rate

Of the 7 RCTs, 5 reported the outcome of complete polyp regression rate^7–10,13^. The anti-VEGF group had a significantly lower complete polyp regression rate than the PDT group (RR: 0.46; 95% CI: 0.28, 0.74), and the early combination group had a significantly higher complete polyp regression rate than the anti-VEGF group (RR: 1.91; 95% CI: 1.40, 2.60). Even though no significant differences were detected between the late and early combination groups, the early combination group exhibited a more favorable trend. Regarding other non-RCTs, 2 of 4 reported this outcome. Li et al. reported no significant differences between the early combination group and anti-VEGF group regarding this outcome. However, Teo et al. reported that the early combination group had a significantly higher complete polyp regression rate than the anti-VEGF group.

### CRT decrease

Seven RCTs reported data of CRT decrease^7–10,13,14,17^. Overall, early combination therapy led to a significantly greater decrease in CRT than did PDT (WMD: 54.83; 95% CI: 12.41, 97.25), anti-VEGF (WMD: 43.40; 95% CI: 19.84, 66.96), or late combination (WMD: − 38.66; 95% CI: − 51.55, 25.77). No significant differences were noted upon comparison of the late combination group with the PDT and anti-VEGF groups. Therefore, early combination therapy might be the optimal treatment modality for reducing CRT. Notably, 2 non-RCTs presented data of CRT decrease. Li et al. reported that early combination therapy caused a more significant decrease in CRT than did anti-VEGF, which is in agreement with the results of our analysis. By contrast, Teo et al. could not detect a significant difference with the same comparison during a 3-month follow-up.

### Total number of anti-VEGF injections

Regarding this outcome, comparisons were only performed among anti-VEGF, early combination, and late combination groups. Overall, 6 of the 7 RCTs reported the total number of anti-VEGF injections during follow-up^7,9,10,13,14,17^. We could not detect a significant difference in the analysis. High heterogenicity (I^2^ = 89.2%) was noted in the comparative analysis between early combination and anti-VEGF groups. The origin of heterogenicity was the study by Lim et al. We speculated that it could be attributable to the small sample size of their study (*n* = 5 in each group). The SUCRA value revealed that late combination therapy might require fewer anti-VEGF injections (SUCRA value 81.9). All non-RCTs presented the data for this outcome. Chong et al. reported that the early combination group required significantly fewer anti-VEGF than did the late combination group. Li et al. reported a significant difference between early combination and anti-VEGF groups. By contrast, Teo et al. observed only a marginally significant difference (*P* = 0.05) with the same comparison. Chen et al. reported that the combination therapy and anti-VEGF groups required a similar number of anti-VEGF injections.

### Safety

#### Systematic adverse events and Ocular adverse events

Of the 7 RCTs, 6 reported data of systematic adverse events^7,9,10,13,14,17^. Overall, no significant difference was detected in this analysis. Regarding ocular adverse events, Five RCTs reported data on the outcome of ocular adverse events^7,10,13,14,17^. The network meta-analysis revealed no significant differences related to ocular adverse events among all treatment modalities.

#### Grading of recommendations, assessment, development, and evaluation

Overall, the certainty of the evidence was low to moderate in our analysis. The level of evidence was downgraded because of the high risk of bias from randomization in most studies as well as indirectness and imprecision. Details are provided in Supplementary 22.

## Discussion

This study is the first systematic review and network meta-analysis of PCV treatments. We included 11 studies published before February 2020 and analyzed 7 RCTs. Notably, the results of the prospective observational studies were heterogenous and not fully compatible with our network meta-analysis of RCTs. Our analysis revealed that early combination therapy might be the optimal therapy in terms of anatomical outcome. However, in terms of BCVA change, anti-VEGF monotherapy was not inferior to the 2 combination therapy modalities.

A significant finding of our study is that the early combination group had the best anatomical outcomes, including complete polyp regression rate and CRT decrease. To our knowledge, anatomical outcomes are the primary focus of PCV treatment; however, the association between polyp closure rate and long-term recurrence rate remains unclear^23^. This finding was different from that of a crucial Fujisan study that reported similar CRT decrease in early and late combination groups^9^. Furthermore, Regarding BCVA change, despite combination therapy exhibiting more favorable trends, our results revealed no significant differences between the 2 modalities of combination therapy and anti-VEGF therapy, which is in agreement with the findings of most studies^7,11,14,16–18^. By contrast, EVEREST II reported that the early combination group had a significantly greater BCVA change than did the anti-VEGF group. This heterogenicity could be attributable to the relatively better baseline BCVA in the population of EVEREST II^10^. Moreover, we noticed that the results of Lim et al.^17^ differed from those of other studies^17^, possibly because of the small sample size (*n* = 10) in their study.

Nevertheless, variations among the population characteristics and treatment protocols merit further exploration and discussion. This information might provide useful insight for clinical practice. We summarized the important information including type of Anti-VEGF agent, protocol of PDT and protocol of rescue therapy in Supplementary 3. Notably, the use of different anti-VEGF agents might be a crucial aspect of PCV treatment. Several studies have reported aflibercept monotherapy to be beneficial in terms of BCVA and anatomical outcome^24,25^. Kawashima et al. reported the effects of aflibercept in ranibizumab-resistant PCV. They indicated a significant difference between BCVA at 6 months and at baseline, albeit with no significant CRT decrease^26^. Recently, Azuma et al. reported the 2-year outcome of treat-and-extend aflibercept for ranibizumab-resistant PCV. They observed a significant CRT decrease from baseline. However, significant BCVA improvement could be observed only at the 1-year mark^27^. Several trials have directly compared the effects of aflibercept and ranibizumab in treating PCV. Notably, even though no significant BCVA change was observed among various anti-VEGF agent groups, the aflibercept group exhibited better anatomical outcomes^28,29^. Among our included RCTs, only one study used aflibercept as the anti-VEGF agent^14^. Nonetheless, because of insufficient data, we could not further compare the effects of aflibercept and ranibizumab with meta-regression or subgroup analysis in our network meta-analysis. Hence, further RCTs are warranted to explore efficacy of different type of anti-VEGF monotherapy and combination therapy.

Because of the recurrent nature of PCV, follow-up time is another critical issue in PCV treatment. A previous systematic review reported that the effect of early PDT could be maintained for 2 years. However, the PCV recurrence rate ranged from 40% to 78.6% after 3 years^23^. Nonetheless, the long-term outcomes of combination therapy were reported recently. Miyata et al. reported that BCVA improved relative to the baseline only in the first year and not after 3 to 5 years. Moreover, CRT after 5 years was reported to be similar to the baseline CRT^30^. Wataru et al. detected a similar deterioration of BCVA improvement after 3 years. However, a significant BCVA improvement was maintained for 5 years. They speculated that the difference between the 2 trials was attributable to the age of their population^31^. The longest follow-up time included in our prospective study was 2 years. Therefore, our analysis could not evaluate long-term changes.

Regarding the safety profile, our study revealed no significant differences related to systematic or ocular adverse events among the therapeutic modalities, and few severe adverse events were reported among these studies. However, several reports have indicated that repeated PDT might damage the retinal pigment epithelium and choriocapillaris layer^32,33^. In addition, Miyata et al. reported a marginally significant increase (*P* = 0.06) in rate of macular atrophy in the combination therapy group, and they considered repeated PDT to be related to macular atrophy in the long term^30^. Nonetheless, there is still no sufficient evidence regarding the long-term consequences of PDT.

### Limitations

Our study had some limitations. First, because of the limited number of RCTs, only 7 RCTs were included in the network meta-analysis. Meta-regression could not be performed for potential confounding factors. Second, CRT measurements reported in the eligible RCTs covered different ranges. Some of them covered the central subfield^10,14^, whereas others only targeted the central fovea. However, no significant inconsistency or serious heterogeneity existed in the pooled estimate of CRT decrease. Third, the follow-up time in most of our included studies was 1 year; therefore, the long-term effects of PCV therapy require evaluation in future studies. Finally, because of much higher incidence and prevalence of PCV in Asian, it was lack of data for other ethnicity. Further trial in other country is warranted for more comprehensive global perspective.

## Conclusions

This study is the first systematic review and network meta-analysis regarding PCV therapy. We recruited prospective observational trials and RCTs. In addition, we critically appraised these studies and performed quantitative analysis of RCTs. Our results revealed that even though no significant differences related to BCVA change were observed among anti-VEGF, late combination, and early combination groups, combination therapy, especially early combination therapy, could result in better anatomical outcomes. Further trials are warranted to investigate the crucial aspects of PCV therapy, such as long-term effects, cost effectiveness, and predictors of therapy response.

## Supplementary Information

Supplementary files
