# Supplementary material for: Systematic review with network meta-analysis of antivascular endothelial growth factor use in managing polypoidal choroidal vasculopathy
Source: Sci Rep. 2021 Feb 2;11:2735. doi: 10.1038/s41598-021-82316-y (PMC7854625; doi:10.1038/s41598-021-82316-y)
Supplement: Supplementary file 1 — Supplementary files [file 41598_2021_82316_MOESM1_ESM.pdf]

Systematic Review With Network Meta-analysis of Antivascular Endothelial Growth Factor Use in Managing Polypoidal Choroidal Vasculopathy

(Appendix File)

Sheng-Chu Chi M.D.+

1. Department of Ophthalmology, Taipei Veterans General Hospital, Taipei, Taiwan Faculty of Medicine, Taipei, Taiwan

Yi-No Kang M.A. +

1. Evidence-Based Medicine Center, Wan Fang Hospital
2. Research center of big data and meta-analysis, Taipei Medical University, Taipei, Taiwan
3. Cochrane Taiwan, Taipei Medical University
4. Institute of Health Policy and Management, College of Public Health, National Taiwan University, Taipei, Taiwan

Yi-Ming Huang M.D*

1. Department of Ophthalmology, Taipei Veterans General Hospital, Taipei, Taiwan Faculty of Medicine
2. National Yang-Ming University School of Medicine, Taipei, Taiwan +

Co-first author: Sheng-Chu Chi M.D. and Yi-No Kang M.A. contribute equally.
*Correspondence: Yi-Ming Huang M.D
Department of Ophthalmology, Taipei Veterans General Hospital, Taipei, Taiwan Faculty of Medicine
E-mail: nowaytokyo@yahoo.com.tw (Huang YM)
Telephone number: +886-2-28757325 Fax number: +886-2-2875

Appendix

Appendix 1. Search strategy

Appendix 2. Flowchart of study selection

Appendix 3. Risk of bias (ROB2 for randomized controlled trials)

Appendix 4. Risk of bias (ROBINS-I for non-randomized controlled trials)

Appendix 5. Publication bias in BCVA change

Appendix 6. Publication bias in BCVA improvement rate

Appendix 7. Publication bias in CRT decrease

Appendix 8. Publication bias in completed polyps regression rate

Appendix 9. Publication bias of Anti-VEGF needed

Appendix 10. Publication bias in systematic adverse Event

Appendix 11. Publication bias in ocular adverse event

Appendix 12. Inconsistency test of BCVA change

Appendix 13. Inconsistency test of BCVA improvement rate

Appendix 14. Inconsistency test of CRT decrease

Appendix 15. Inconsistency test of completed polyps regression rate

Appendix 16. Inconsistency test of Anti-VEGF needed

Appendix 17. Inconsistency test of systematic adverse Event

Appendix 18. Inconsistency test of ocular adverse event

Appendix 19. SUCRA of BCVA change

Appendix 20. SUCRA of BCVA improvement rate

Appendix 21. SUCRA of BCVA Anti-VEGF needed

Appendix 22. GRADE

Appendix 1
Search strategy

Appendix 1
Search strategy

Primary search steps:

#1 polypoidal choroidal vasculopathy
#2. PCV
#3. 1 or 2
#4. photodynamic therapy
#5. PDT
#6. #4 or #5
#7. anti- vascular endothelial growth factors
#8. anti-VEGF
#9. ranibizumab
#10. lucentis
#11. aflibercept
#12. eylea
#13. bevacizumab,
#14. avastin
#15. 7 or 8 or 9 or 10 or 11 or 12 or 13 or 14
#16. 3 and 6 and 15

Final syntax in PubMed (an example):

(((Polypoidal choroidal vasculopathy OR PCV OR polypoidal choroidal neovascularisation OR polypoidal choroidal neovascularization OR CNV OR PCNV)) AND (photodynamic therapy OR PDT OR photosensitization OR photosensitiz* OR photosensitisation OR photochemotherapy OR chemophototherapy)) AND (anti-vascular endothelial growth factors OR anti-vascular endothelial growth factor OR anti-VEGF OR ranibizumab OR lucentis OR aflibercept OR Eylea OR Zaltrap OR ziv-aflibercept OR VEGF trap OR Bevacizumab OR Avastin OR zirabev)

Appendix 2
Flowchart of study selection

Appendix 2

Flowchart of study selection

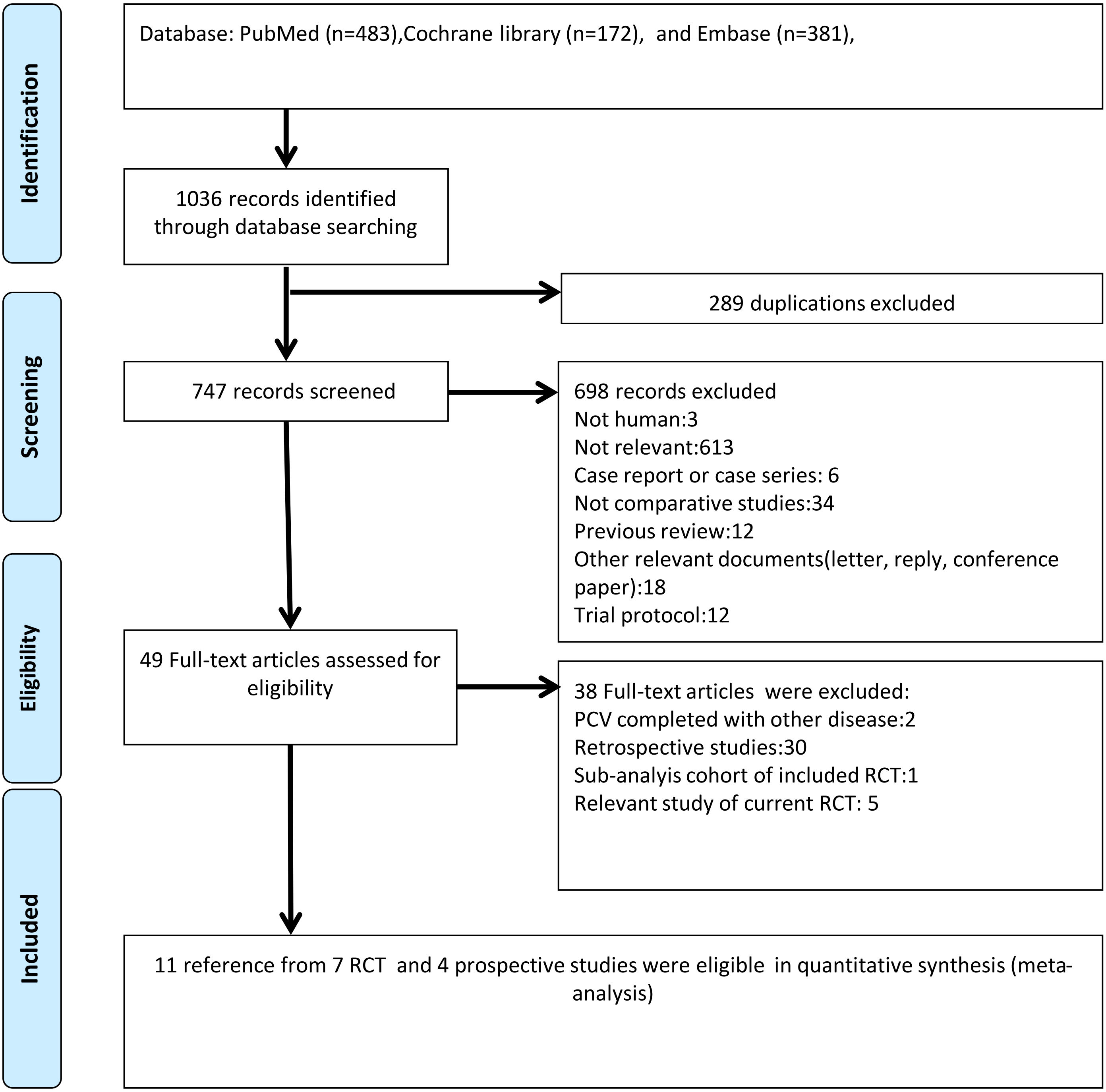

Appendix 3 to 4
Risk of bias

Appendix 3

Risk of bias (ROB2 for randomized controlled trials)

Outcome/ Study	Bias due to: Randomization process	Deviations from intended interventions	Missing outcome data	Measurement of the outcome	Selection of the reported result	Overall bias
**BCVA change**						**SC**
EVEREST	SC [a,b]	Low	Low	Low[d]	Low	
EVEREST II	SC [b]	SC[c]	Low	Low	Low	
Fujisan Trial	SC [a,b]	Low	Low	Low[d]	Low	
Lai et al. (2018)	SC [a,b]	Low	Low	Low[d]	Low	
Lim et al (2012)	SC [a,b]	Low	Low	Low[d]	Low	
Oishi et al. (2013)	SC [a,b]	SC[c]	Low	Low[d]	Low	
PLANET	Low	SC[c]	Low	Low	Low	
**BCVA improvement rate**						**SC**
EVEREST	SC [a,b]	Low	Low	Low[d]	Low	
EVEREST II	SC [b]	SC[c]	Low	Low	Low	
Lai et al. (2018)	SC [a,b]	Low	Low	Low[d]	Low	
Oishi et al. (2013)	SC [a,b]	SC[c]	Low	Low[d]	Low	
PLANET	Low	SC[c]	Low	Low	Low	
**CRT decrease**						**SC**
EVEREST	SC [a,b]	Low	Low	Low[d]	Low	
Fujisan Trial	SC [a,b]	Low	Low	Low[d]	Low	
Lai et al. (2018)	SC [a,b]	Low	Low	Low[d]	Low	
Lim et al (2012)	SC [a,b]	Low	Low	Low[d]	Low	
Oishi et al. (2013)	SC [a,b]	SC[c]	Low	Low[d]	Low	
**Completed polyp regression**						**SC**
EVEREST	SC [a,b]	Low	Low	Low[d]	Low	
EVEREST II	SC [b]	SC[c]	Low	Low	Low	
Fujisan Trial	SC [a,b]	Low	Low	Low[d]	Low	
Lai et al. (2018)	SC [a,b]	Low	Low	Low[d]	Low	
PLANET	Low	SC[c]	Low	Low	Low	
**Number of Anti-VEGF**						**SC**
EVEREST	SC [a,b]	Low	Low	Low[d]	Low	
EVEREST II	SC [b]	SC[c]	Low	Low	Low	
Fujisan Trial	SC [a,b]	Low	Low	Low[d]	Low	
Lai et al. (2018)	SC [a,b]	Low	Low	Low[d]	Low	
Lim et al (2012)	SC [a,b]	Low	Low	Low[d]	Low	
Oishi et al. (2013)	SC [a,b]	SC[c]	Low	Low[d]	Low	
**Adverse event**						**SC**
EVEREST	SC [a,b]	Low	Low	Low[d]	Low	
EVEREST II	SC [b]	SC[c]	Low	Low	Low	
Fujisan Trial	SC [a,b]	Low	Low	Low[d]	Low	
Lai et al. (2018)	SC [a,b]	Low	Low	Low[d]	Low	
Lim et al (2012)	SC [a,b]	Low	Low	Low[d]	Low	
PLANET	Low	SC[c]	Low	Low	Low	
**Ocular adverse event**						**SC**
EVEREST	SC [a,b]	Low	Low	SC[e]	Low	
EVEREST II	SC [b]	SC[c]	Low	Low	Low	
Fujisan Trial	SC [a,b]	Low	Low	Low[d]	Low	
Lai et al. (2018)	SC [a,b]	Low	Low	Low[d]	Low	
Lim et al (2012)	SC [a,b]	Low	Low	Low[d]	Low	
PLANET	Low	SC[c]	Low	Low	Low	

SC, some concerns

a. no information about the concealment of allocation sequence

b. no information about the random method

c. deviations from the intended intervention arose because of the experimental context, but not affect outcome

d. outcome assessors aware of the intervention, but assessment of the outcome was not likely be influenced

e. outcome assessors aware of the intervention, and assessment of the outcome was likely be influenced by accessors judgement

Appendix 4

Risk of bias (ROBINS-I for non-randomized controlled trials)

/ Study	Bias due to						Overall bias
	Cofounding	Selection	Measurement of intervention	Missing data	Measurement of outcome	Reported result	
Chen et al	Serious	Low	Moderate	Moderate	Low	Moderate	Serious
Chong et al	Moderate	Low	Low	Low	Low	Low	Moderate
Li et al	Moderate	Low	Low	Low	Low	Moderate	Moderate
Teo et al	Moderate	Low	Low	Low	Low	Low	Moderate

Appendix 5 to 11
Publication bias

Appendix 5

Publication bias in BCVA change

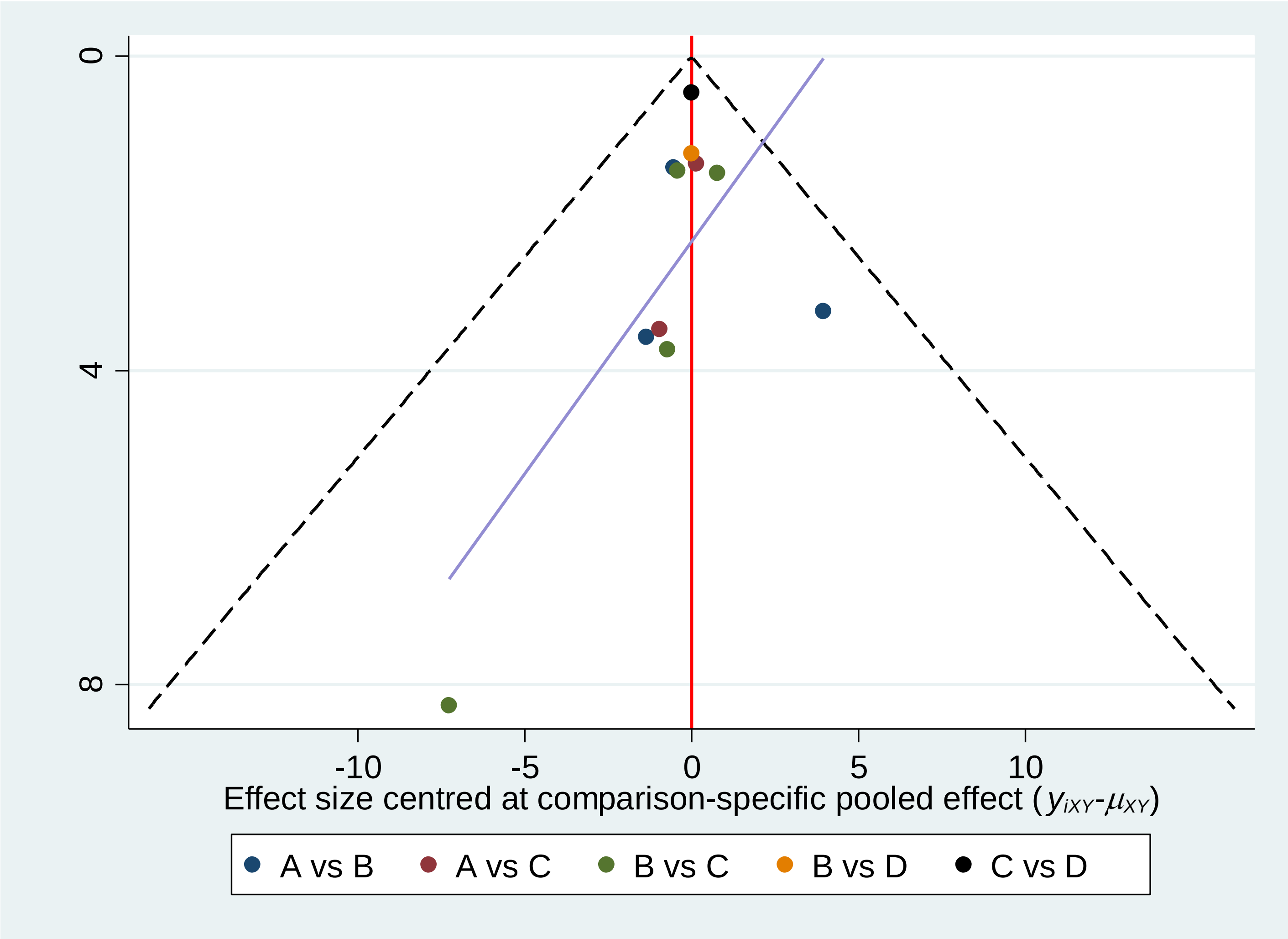

Egger's test for small-study effects:

Number of studies = 11 Root MSE = .5736

Std_Eff	Coef.	Std. Err.	t	P>|t|	[95% Conf.	Interval]
slope	.1103576	.3259109	0.34	0.743	-.6269041	.8476194
bias	-.1191229	.2704775	-0.44	0.670	-.7309854	.4927397

Appendix 6

Publication bias in BCVA improvement rate

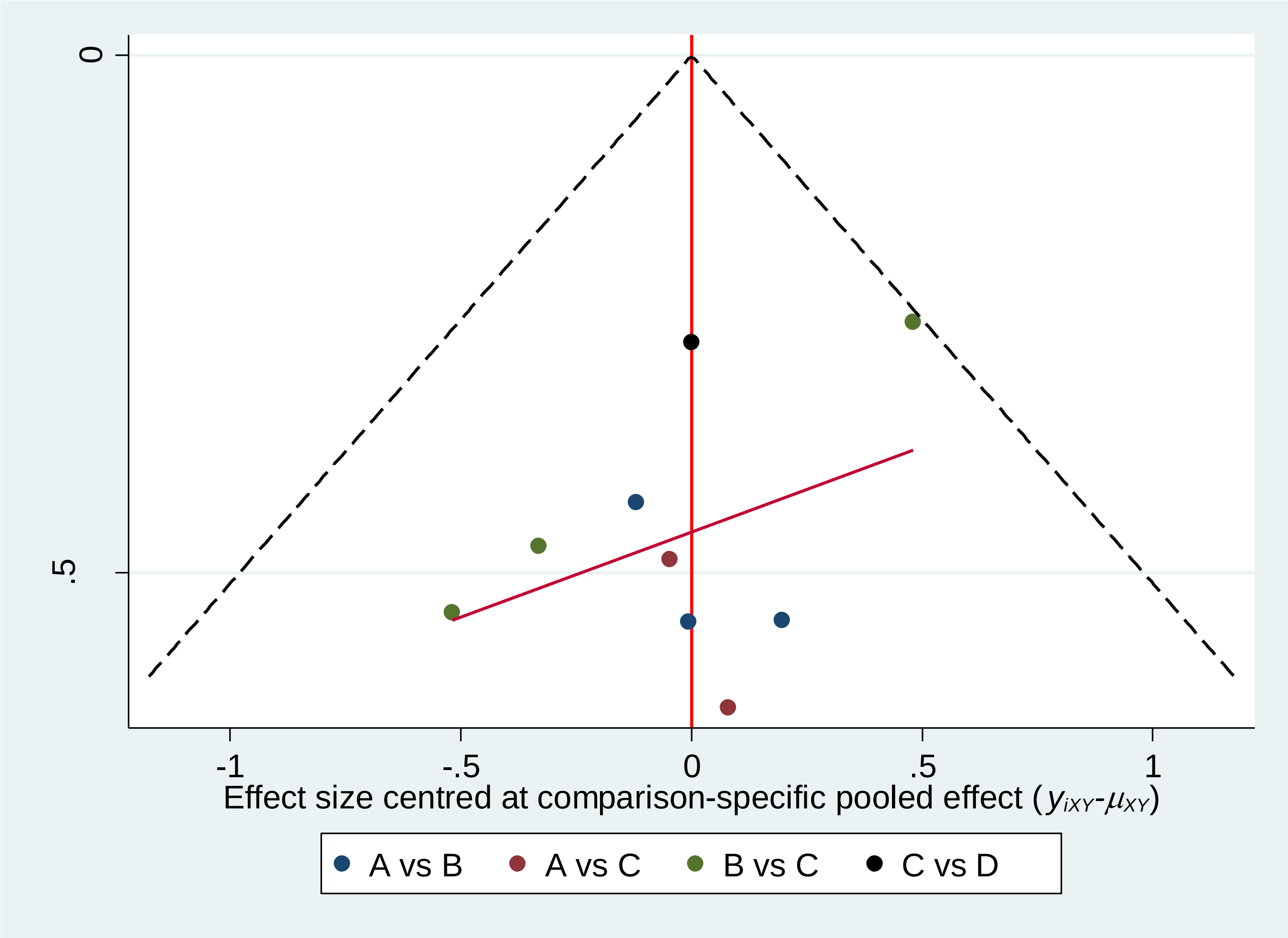

Egger's test for small-study effects:

Number of studies = 9   Root MSE = .6708

Std_Eff	Coef.	Std. Err.	t	P>|t|	[95% Conf.	Interval]
slope	.5995948	.287573	2.09	0.076	-.0804072	1.279597
bias	-1.370244	.7094823	-1.93	0.095	-3.047903	.3074152

Appendix 7

Publication bias in CRT decrease

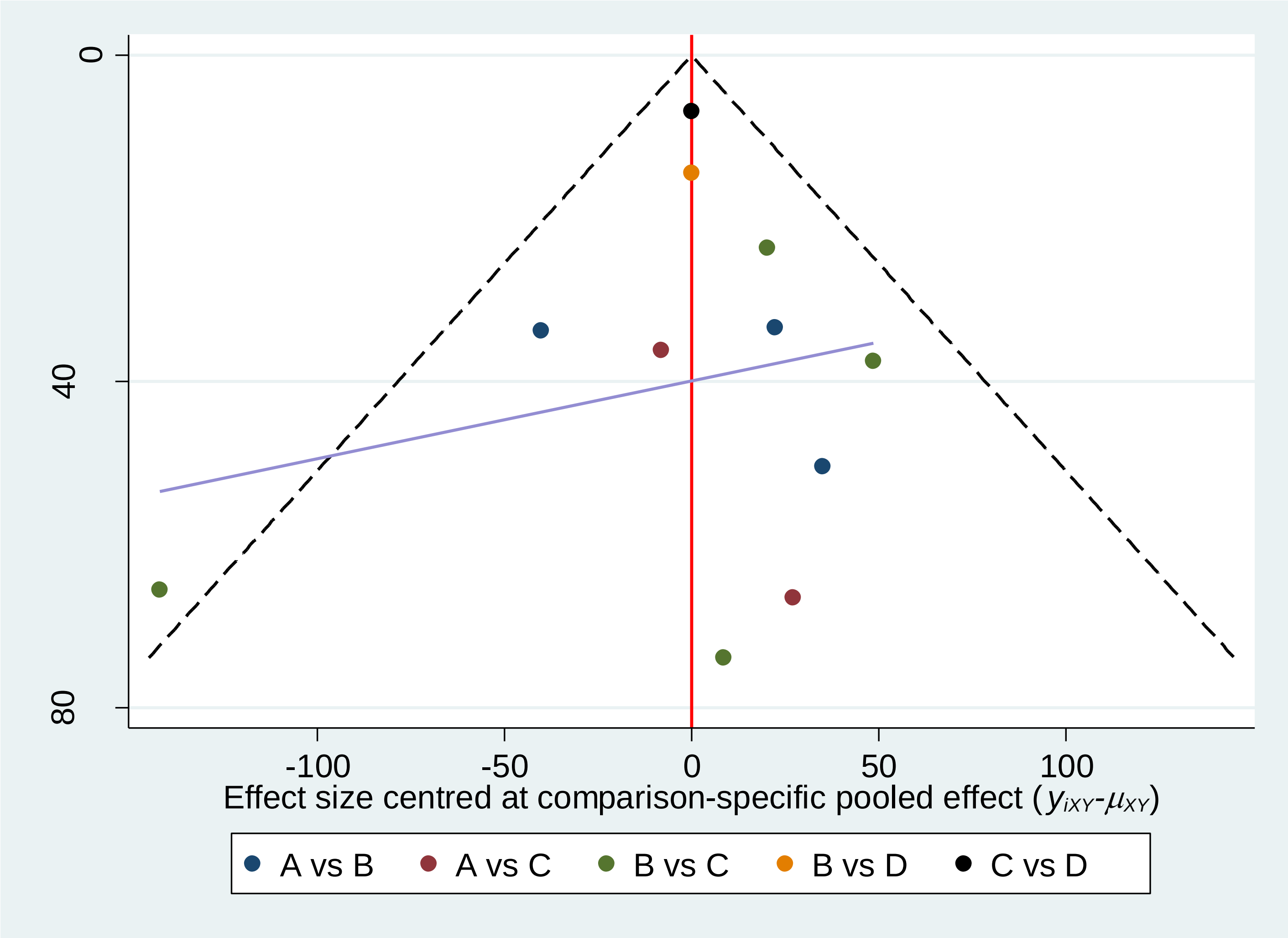

Egger's test for small-study effects:

Number of studies = 11 Root MSE = 1.033

Std_Eff	Coef.	Std. Err.	t	P>|t|	[95% Conf.	Interval]
slope	1.308397	8.450438	0.15	0.880	-17.80782	20.42462
bias	-.0101359	.4566048	-0.02	0.983	-1.043048	1.022776

Appendix 8

Publication bias in completed polyps regression rate

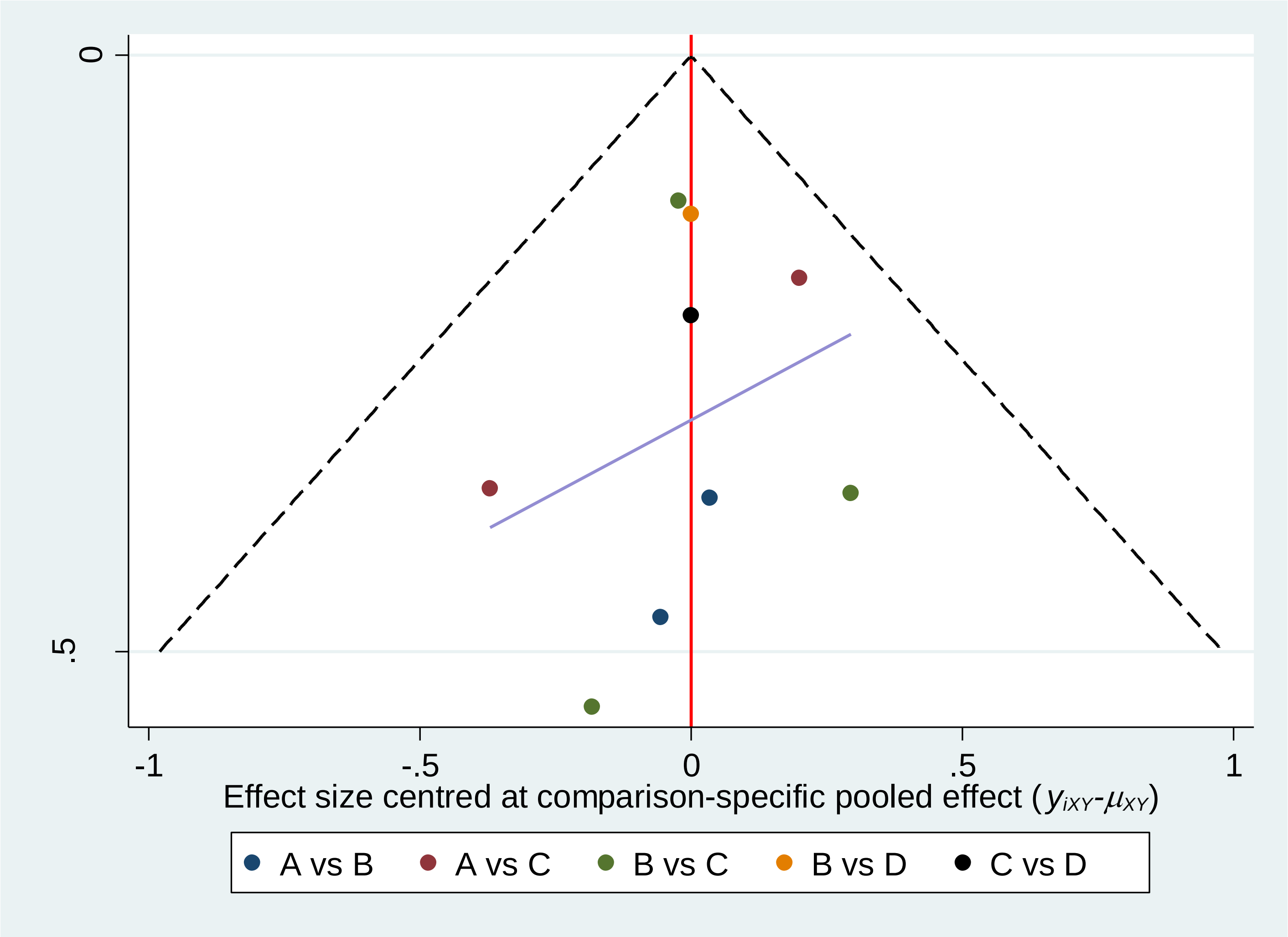

Egger's test for small-study effects:

Number of studies = 9 Root MSE = .6463

Std_Eff	Coef.	Std. Err.	t	P>|t|	[95% Conf.	Interval]
slope	.0366368	.0963995	0.38	0.715	-.1913119	.2645854
bias	-.1202725	.4582782	-0.26	0.801	-1.203928	.9633833

Appendix 9
Publication bias in Anti-VEGF needed

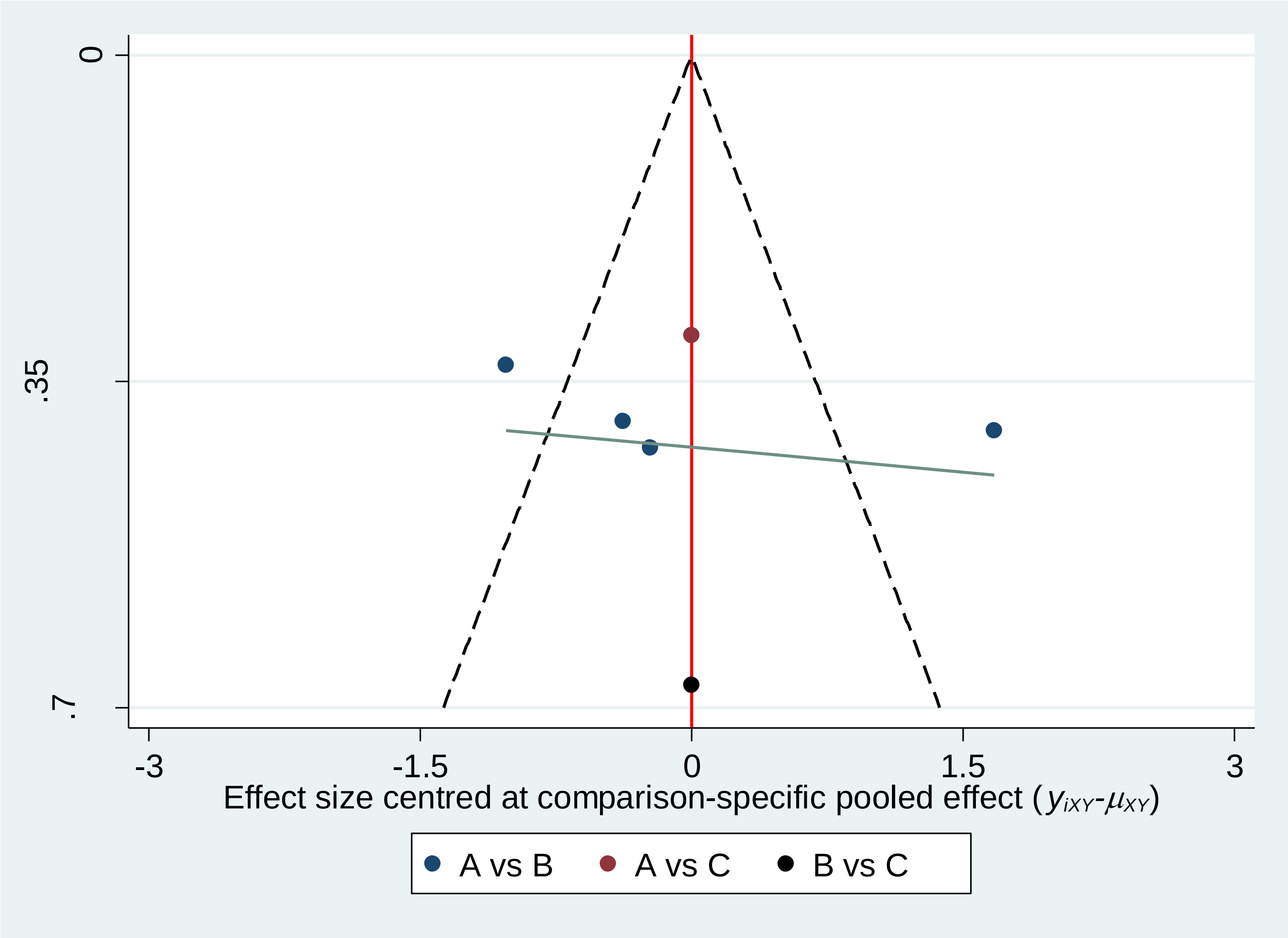

Egger's test for small-study effects:

Number of studies = 6   Root MSE = 2.585

Std_Eff	Coef.	Std. Err.	t	P>|t|	[95% Conf. Interval]	
slope	-.8041235	1.827873	-0.44	0.683	-5.879111	4.270864
bias	1.966396	4.755861	0.41	0.700	-11.23799	15.17078

Appendix 10

Publication bias in systematic adverse event

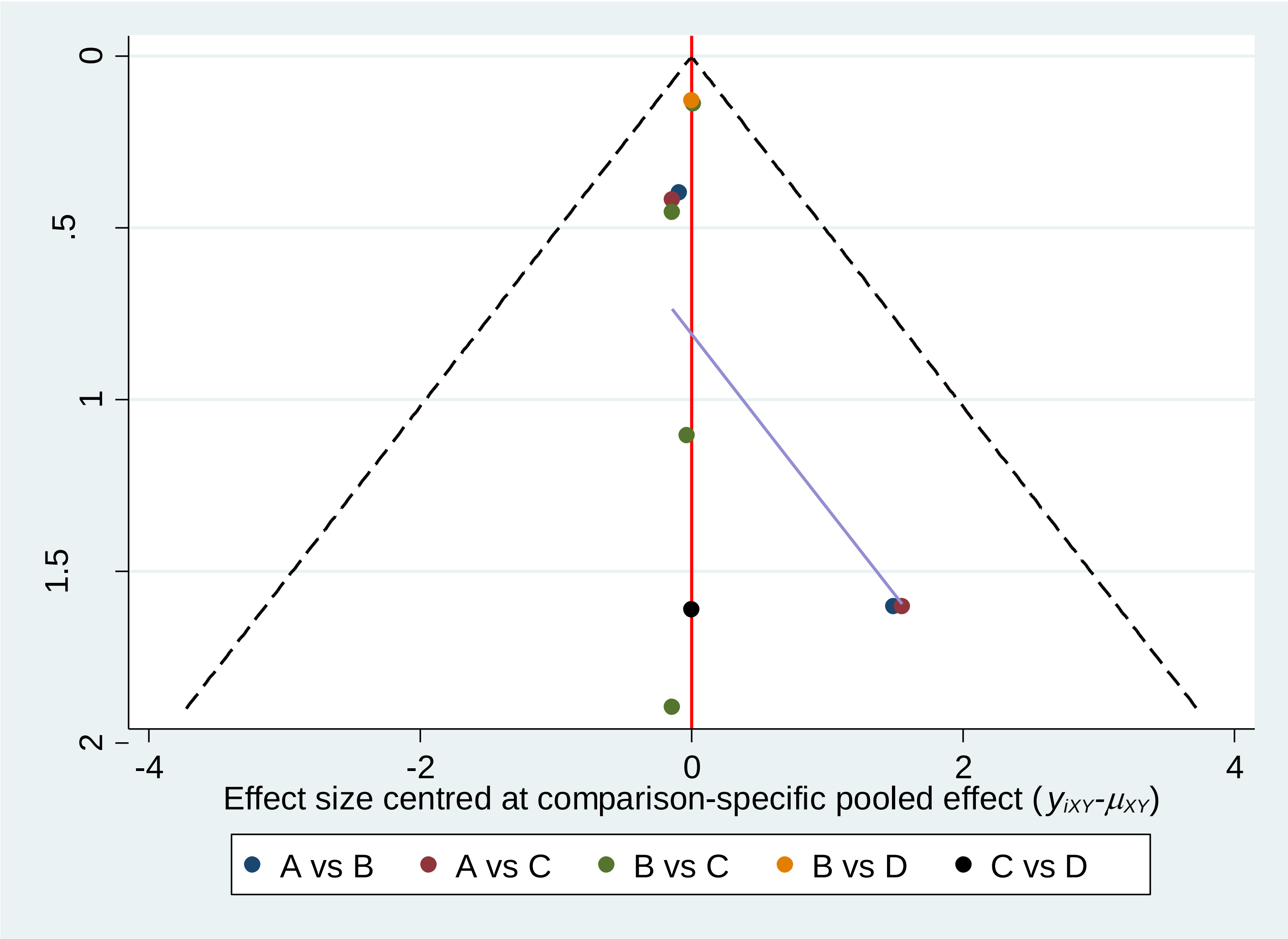

Egger's test for small-study effects:

Number of studies = 10 Root MSE = .484

Std_Eff	Coef.	Std. Err.	t	P>|t|	[95% Conf.	Interval]
slope	-.0411854	.0589037	-0.70	0.504	-.1770175	.0946468
bias	.2059532	.2141788	0.96	0.364	-.2879439	.6998503

Appendix 11
Publication bias in ocular adverse event

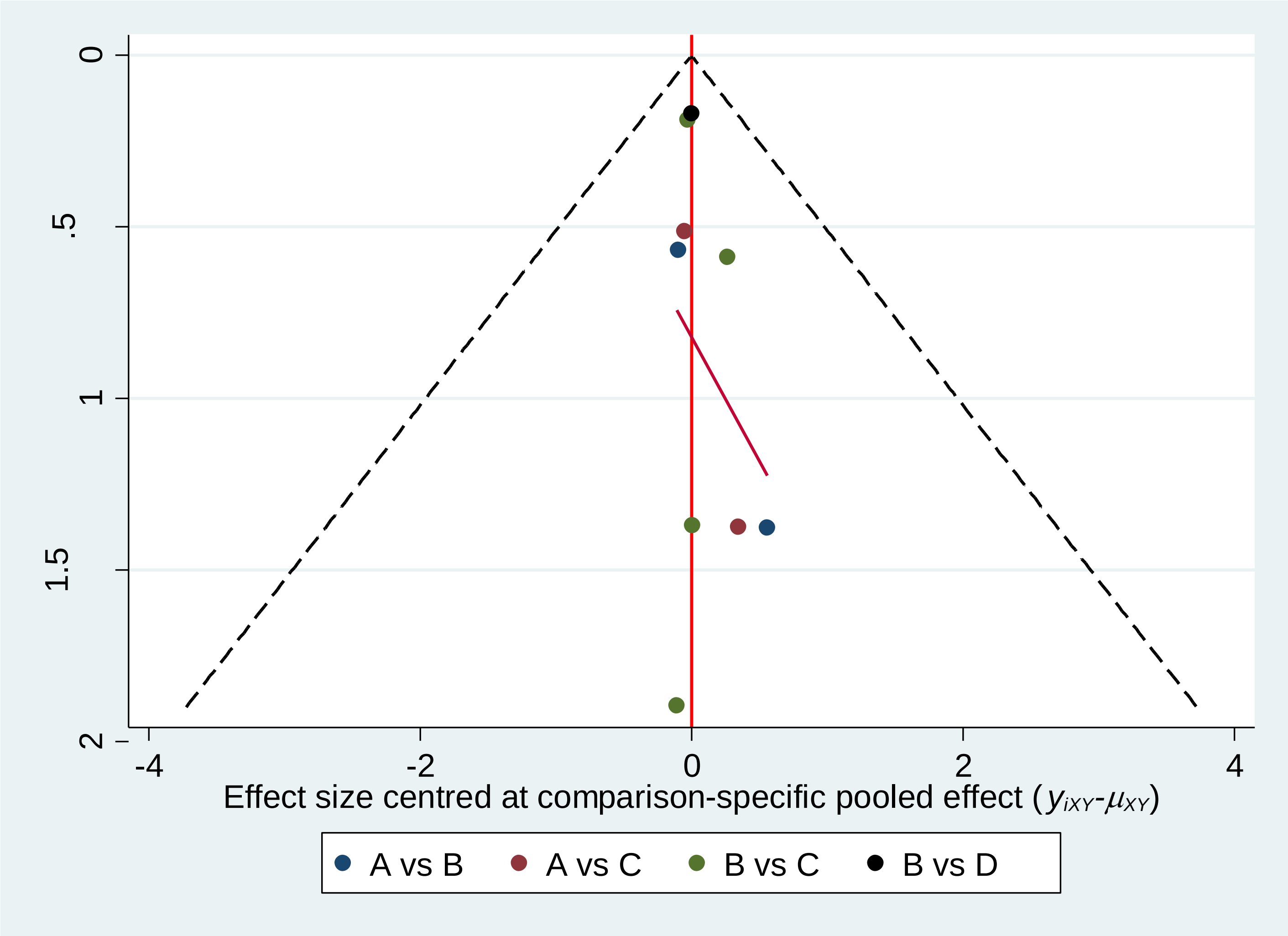


```
                 Egger's test for small-study effects:
Number of studies =  9                                 Root MSE      =   .2336
------------------------------------------------------------------------------
     Std_Eff |      Coef.   Std. Err.      t    P>|t|     [95% Conf. Interval]
-------------+----------------------------------------------------------------
       slope |  -.0426496   .0407506    -1.05   0.330    -.1390095    .0537102
        bias |     .16406   .1170054     1.40   0.204    -.1126138    .4407338
------------------------------------------------------------------------------
```

Appendix 12 to 18
Inconsistency test

Appendix 12

Inconsistency test of BCVA change

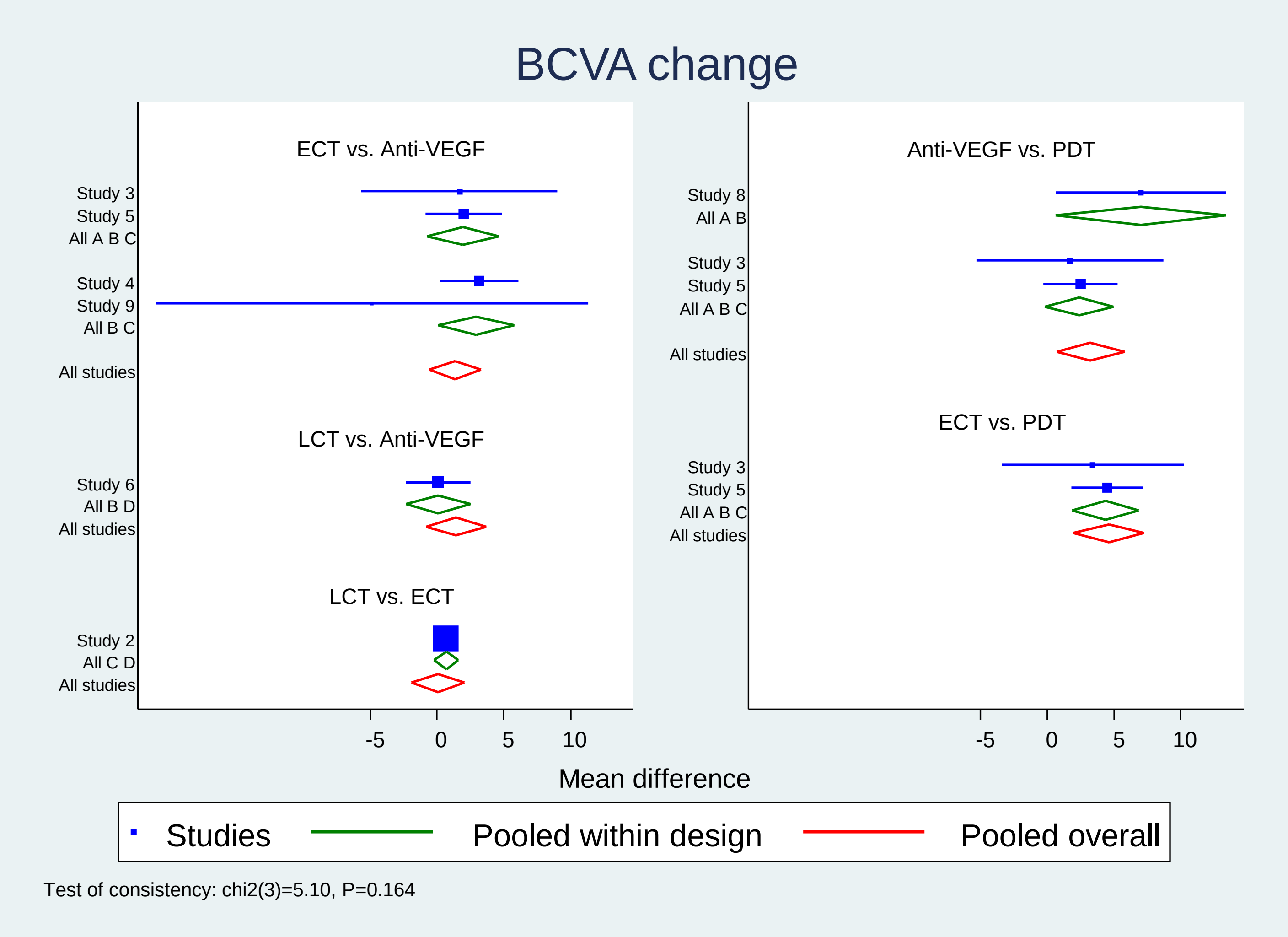

Appendix 13

Inconsistency test of BCVA improvement rate

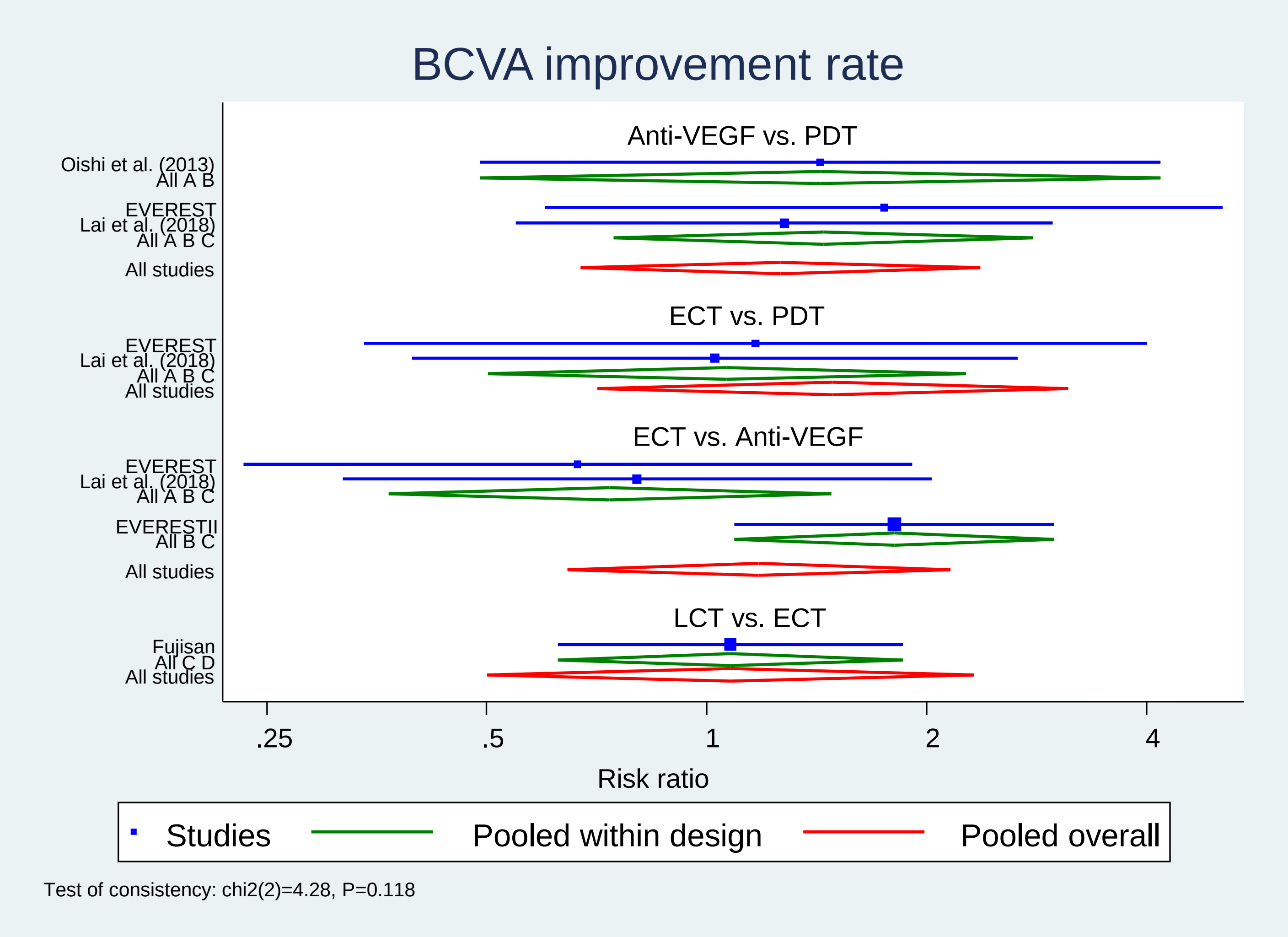

Appendix 14

Inconsistency test of completed polyps regression rate

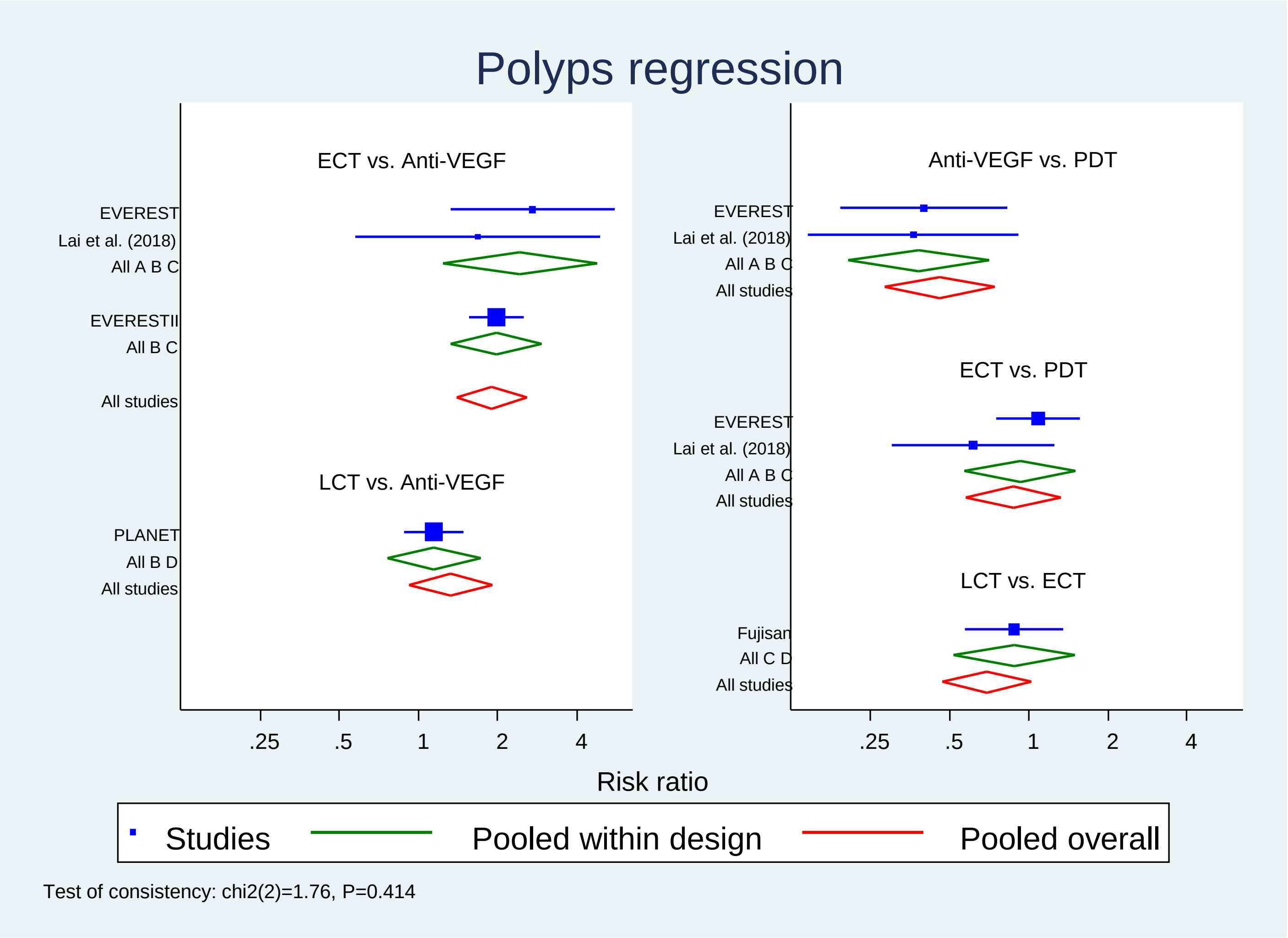

Appendix 15

Inconsistency test of CRT decrease

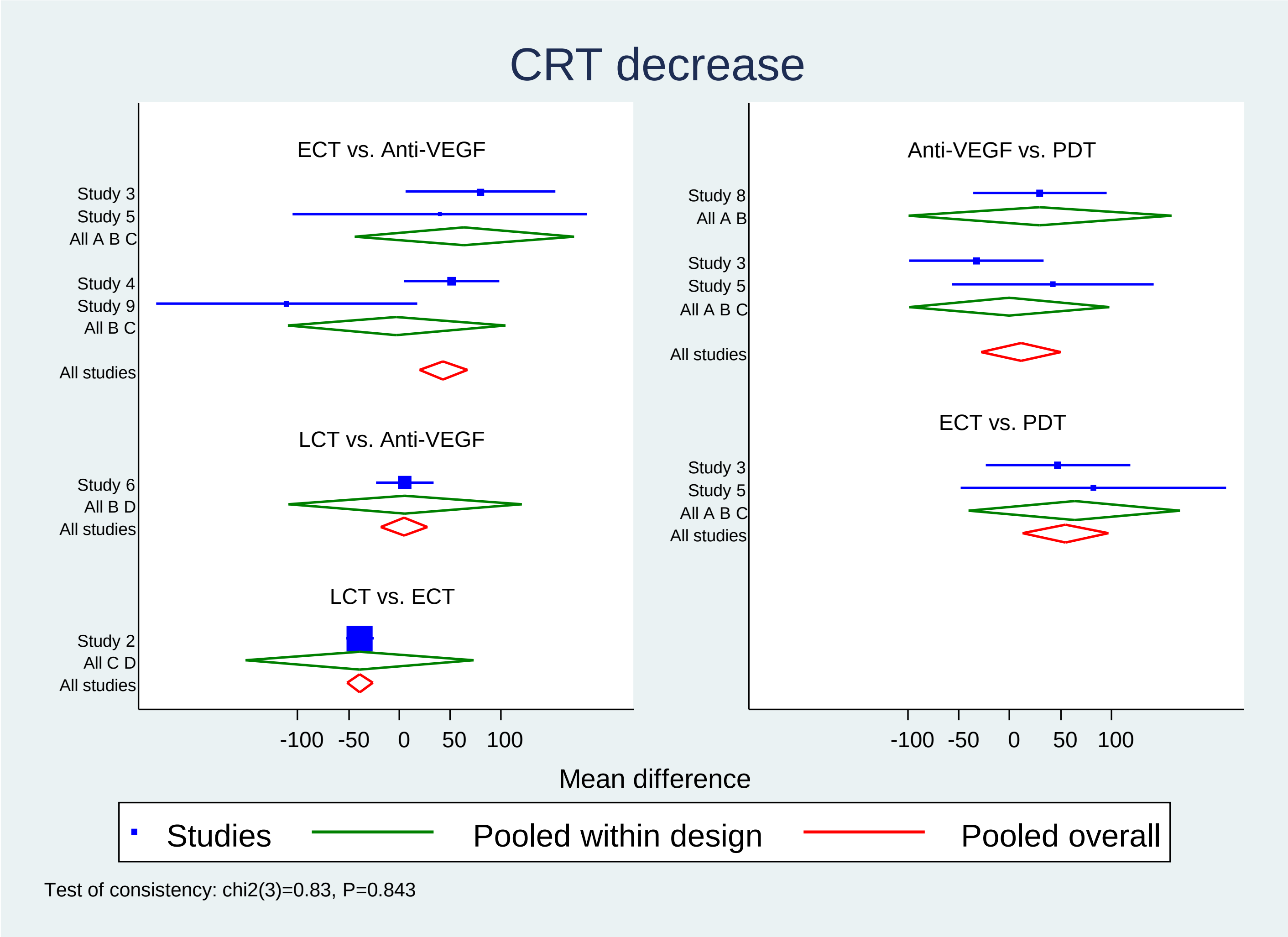

Appendix 16

Inconsistency test of Anti-VEGF needed

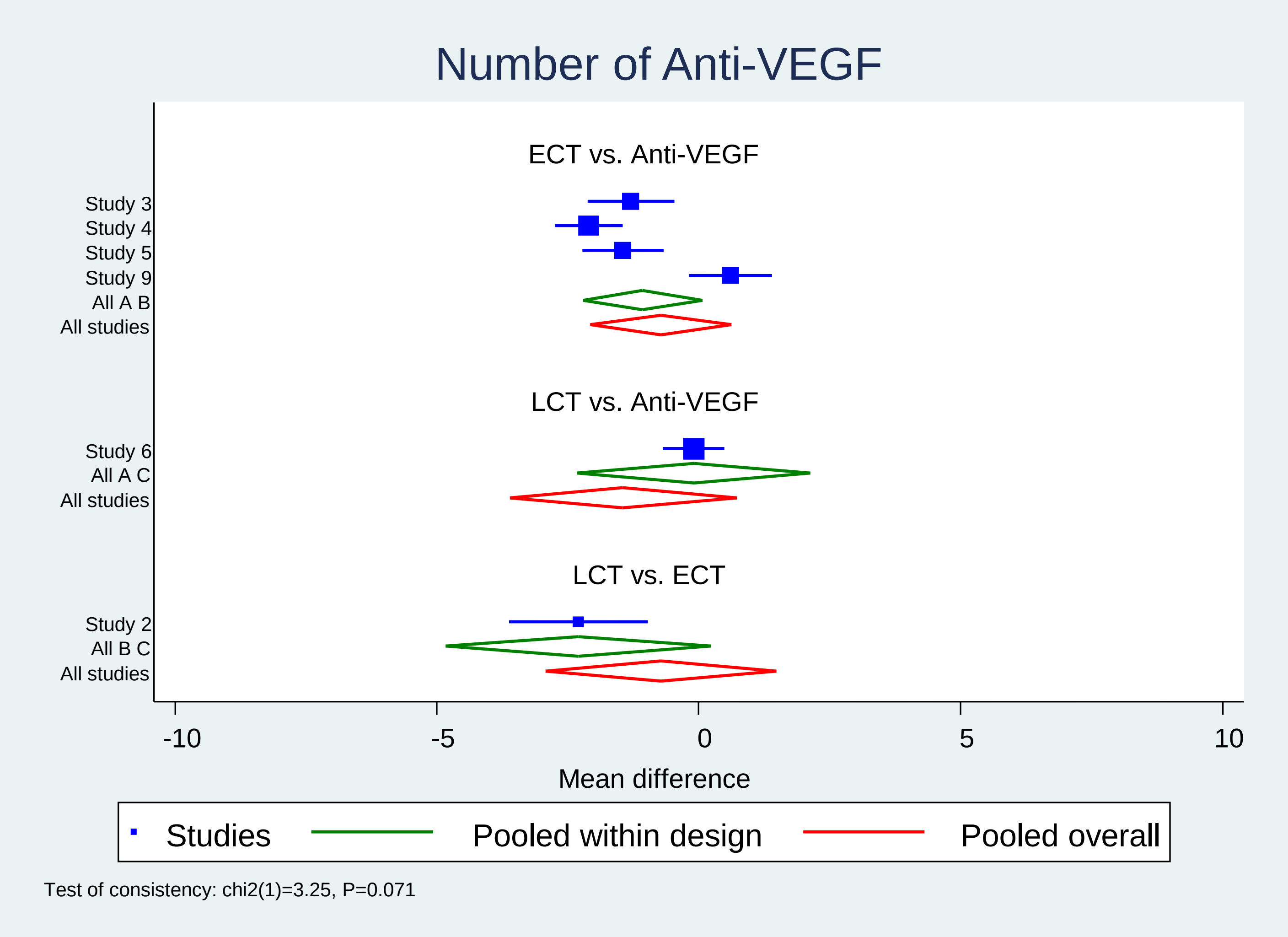

Appendix 17

Inconsistency test of systematic adverse event

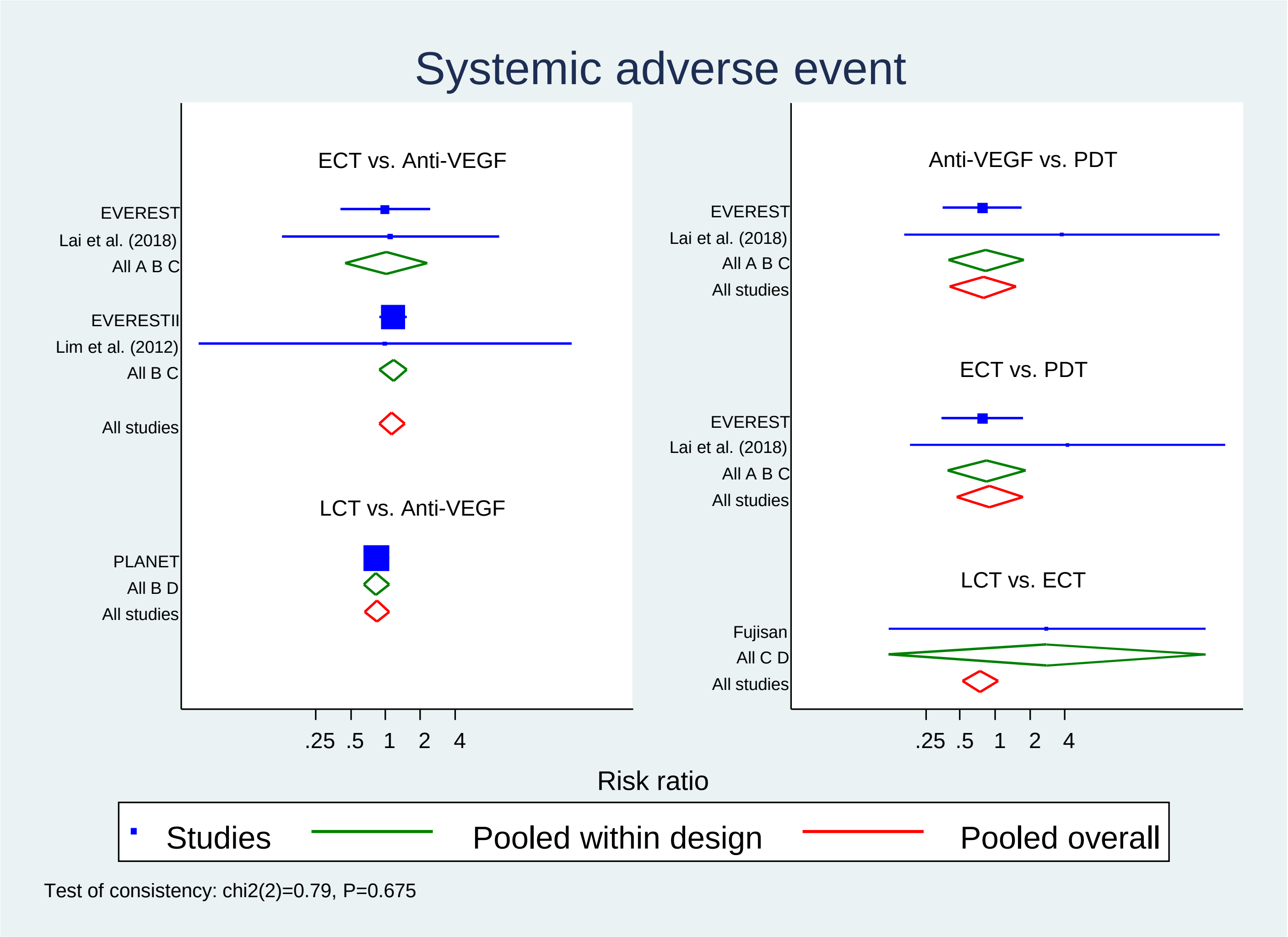

Appendix 18

Inconsistency test of ocular adverse event

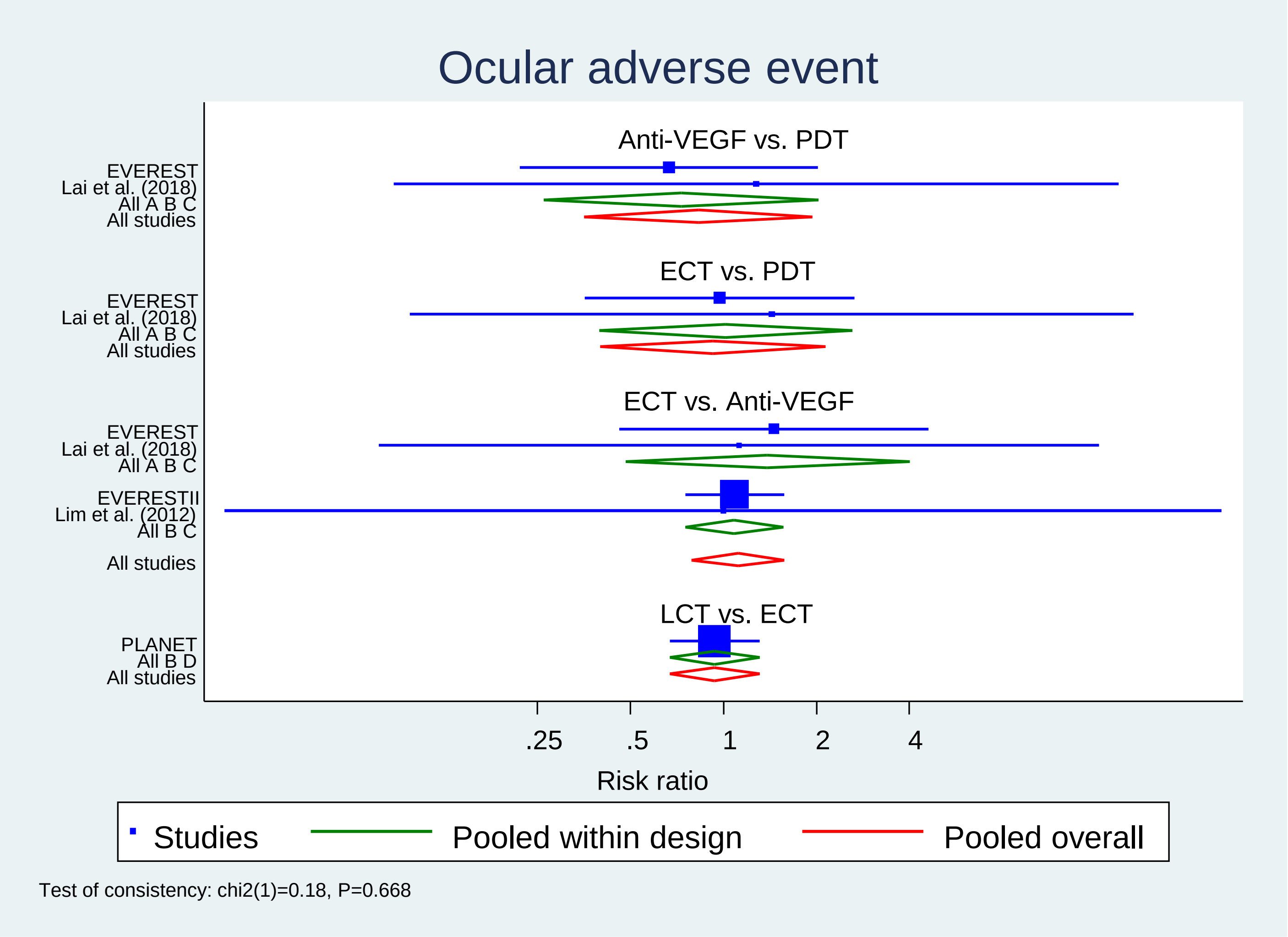

Appendix 19 to 21
SUCRA

Appendix 19
SUCRA of BCVA change

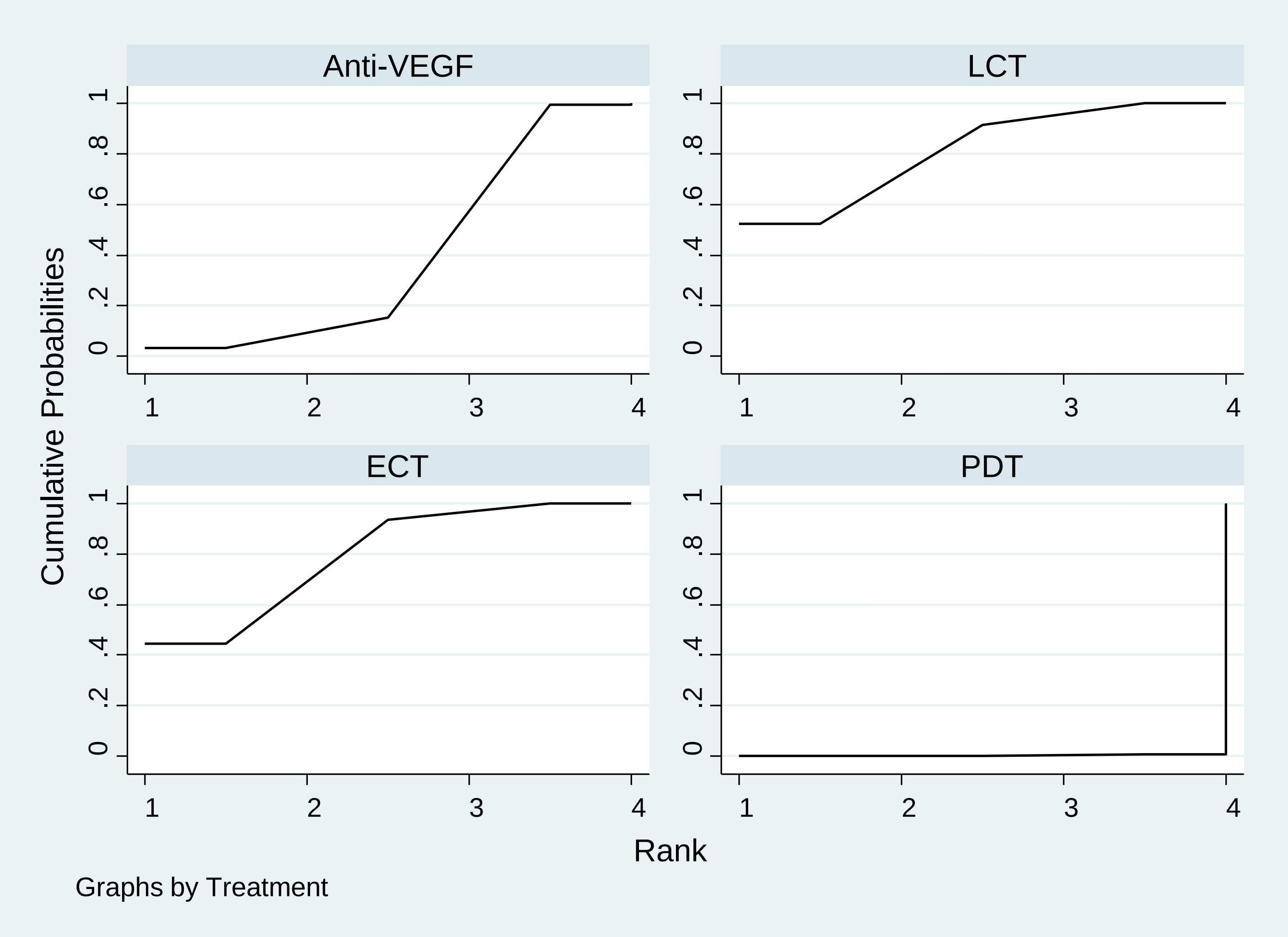

Treatment	SUCRA	PrBest	MeanRank
PDT	0.2	0.0	4.0
Anti-VEGF	39.2	3.1	2.8
ECT	79.4	44.6	1.6
LCT	81.2	52.3	1.6

Appendix 20
SUCRA of BCVA improvement rate

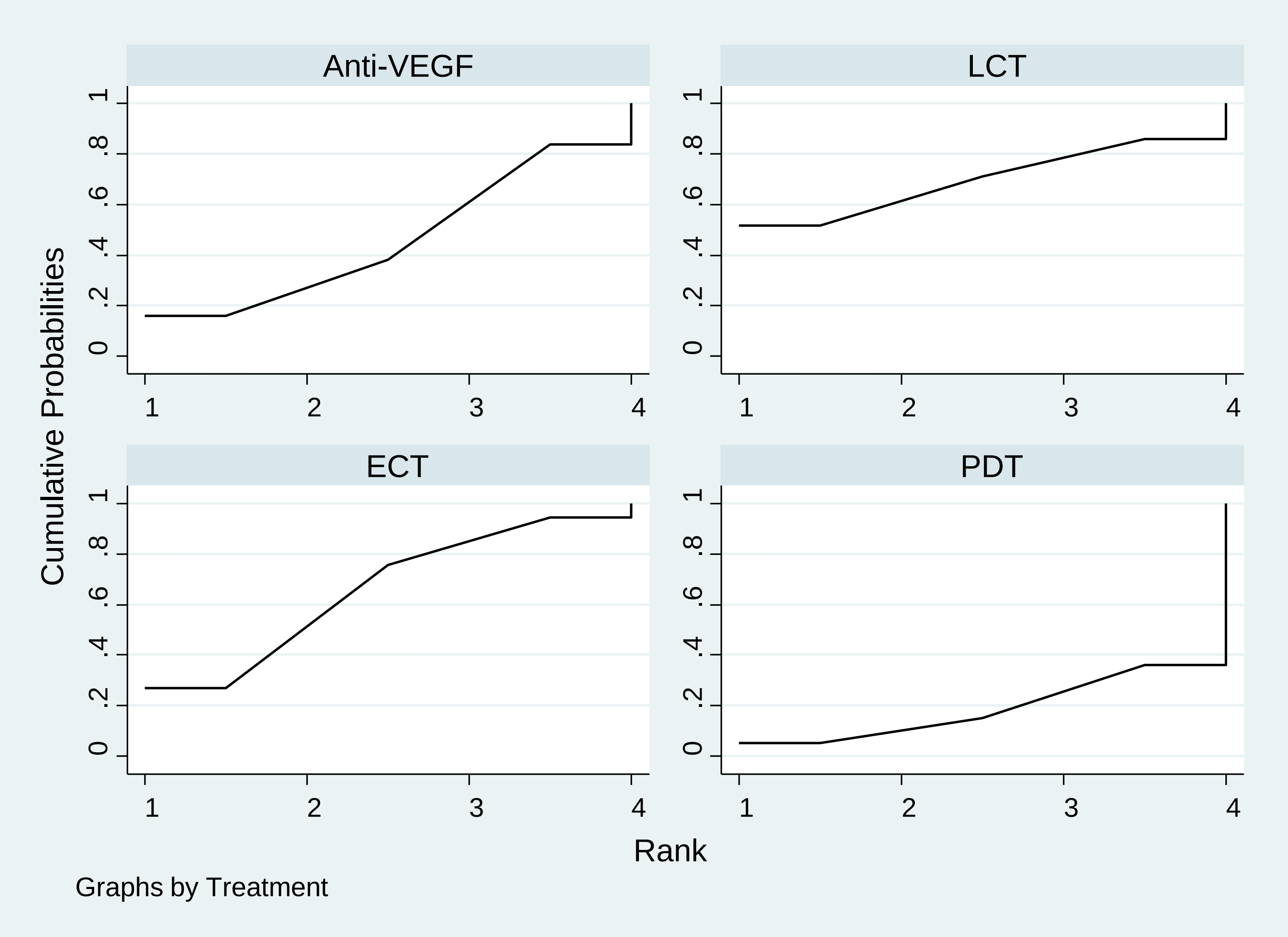

Treatment	SUCRA	PrBest	MeanRank
PDT	19.0	5.5	3.4
Anti-VEGF	45.9	15.9	2.6
ECT	65.6	27.0	2.0
LCT	69.4	51.6	1.9

Appendix 21
SUCRA of Anti-VEGF needed

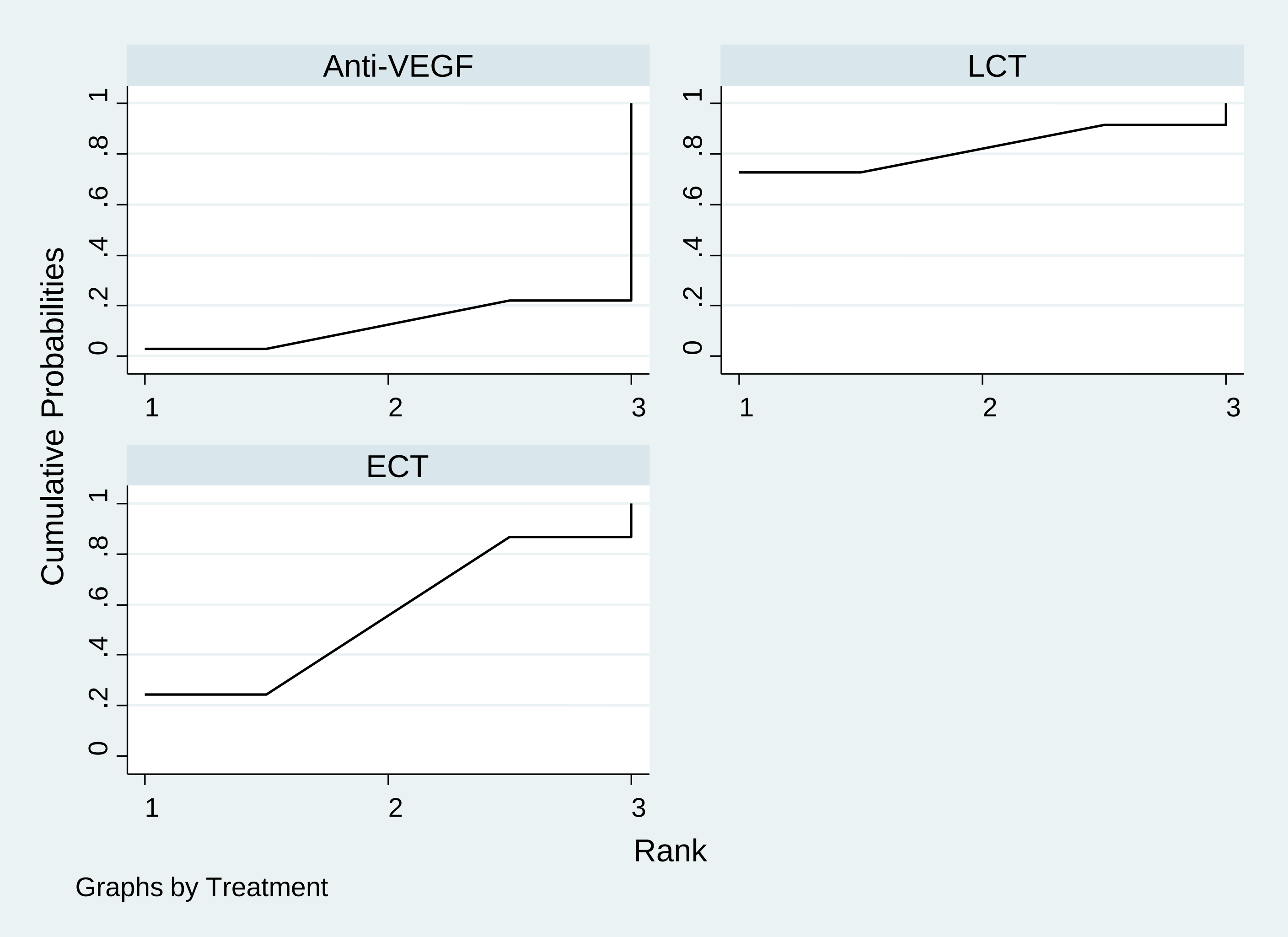

Treatment	SUCRA	PrBest	MeanRank
Anti-VEGF	12.4	2.8	2.8
ECT	55.7	24.5	1.9
LCT	81.9	72.6	1.4

Appendix 22
GRADE

Appendix 22

GRADE

PDT VS Anti-VEGF VS Early combination VS Late combination for PCV-BCVA change

Certainty assessment							Summary of findings	
							Anticipated absolute effects	
№ of eyes (studies) Follow-up	Risk of bias	Inconsistency	Indirectness	Imprecision	Publication bias	Overall certainty of evidence	Effect of control	Effect difference with intervention
**BCVA change- PDT VS Anti-VEGF**								
176 (3 RCTs)	serious [a,b]	not serious [c]	not serious	not serious	none	⊕⊕⊕◯ MODERATE	The mean BCVA changed ranged from **-2.50 to 7.50 letters**	WMD **3.22 higher** (0.68 higher to 5.76 higher)
**BCVA change- PDT VS Early combination**								
78 (2 RCTs)	serious [a,b]	not serious	not serious	not serious	none	⊕⊕⊕◯ MODERATE	The mean BCVA changed ranged from **7.50 to 7.50 letters**	WMD **4.59 higher** (1.95 higher to 7.22 higher)
**BCVA change- PDT VS Late combination**								
AIC	serious [a,b]	not serious	serious	not serious	none	⊕⊕◯◯ LOW	The mean BCVA changed ranged from **-2.50 to 7.50 letters**	WMD **4.67 higher** (1.68 higher to 7.67 higher)
**BCVA change- Anti-VEGF VS Early combination**								
401 (4 RCTs)	serious [a,b]	not serious	not serious	not serious	none	⊕⊕⊕◯ MODERATE	The mean BCVA changed ranged from **5.10 to 10.00 letters**	WMD **1.37 higher** (0.57 lower to 3.37 higher)
**BCVA change- Anti-VEGF VS Late combination**								
318 (1 RCTs)	not serious	not serious	not serious	not serious	none	⊕⊕⊕⊕ High	The mean BCVA changed ranged from **10.70 to 10.70 letters**	WMD **1.45 higher** (0.77 lower to 3.68 higher)
**BCVA change- Early combination VS Late combination**								
60 (1 RCTs)	serious [a,b]	not serious	not serious	not serious	none	⊕⊕⊕◯ MODERATE	The mean BCVA changed ranged from **5.10 to 5.10 letters**	WMD **0.08 higher** (1.91 lower to 2.07 higher)

PDT VS Anti-VEGF VS Early combination VS Late combination for PCV-CRT decrease

Certainty assessment							Summary of findings	
							Anticipated absolute effects	
№ of participants (studies) Follow-up	Risk of bias	Inconsistency	Indirectness	Imprecision	Publication bias	Overall certainty of evidence	Effect of control	Effect difference with intervention
**CRT decrease- PDT VS Anti-VEGF**								
176 (3 RCTs)	serious [a,b]	not serious	not serious	not serious	none	⊕⊕⊕◯ MODERATE	The mean CRT decrease ranged from **77.7 to 111.61um**	WMD **12.38 higher** (26.50 lower to 51.25 higher)
**CRT decrease- PDT VS Early combination**								
78 (2 RCT)	serious [a,b]	not serious [f]	not serious	not serious	none	⊕⊕⊕◯ MODERATE	The mean CRT decrease ranged from **98.1 to 111.61um**	WMD **52.20 higher** (12.30 higher to 92 higher)
**CRT decrease - PDT VS Anti-VEGF+ Post-PDT**								
AIC	serious [a,b]	not serious	serious	not serious	none	⊕⊕◯◯ LOW	The mean CRT decrease ranged from **77.7 to 111.61um**	WMD **16.17 higher** (26.44 lower to 58.78 higher
**CRT decrease- Anti-VEGF VS Early combination**								
405 (4 RCTs)	serious [a,b]	not serious	not serious	not serious	none	⊕⊕⊕◯ MODERATE	The mean CRT decrease ranged from **65.7 to 154.33 um**	WMD **43.40 higher** (19.84 higher to 66.96 higher)
**CRT decrease- Anti-VEGF VS Anti-VEGF+ Post-PDT**								
318 (1 RCT)	not serious	not serious [f]	not serious	not serious	none	⊕⊕⊕⊕ High	The mean CRT decrease ranged from **137.7 to 137.7um**	WMD **4.74 higher** (18.01 lower to 27.48 higher)
**CRT decrease - Early combination VS Anti-VEGF+ Post-PDT**								
60 (1 RCTs)	serious [a,b]	not serious	not serious	not serious	none	⊕⊕⊕◯ MODERATE	The mean CRT decrease ranged from **184.5 to 184.5 um**	WMD **38.66 lower** (51.55 lower to 25.77 lower)

PDT VS Anti-VEGF VS Early combination VS Late combination for PCV-complete polyp regression rate

Certainty assessment							Summary of findings				
							Study event rates (%)			Anticipated absolute effects	
№ of participants (studies) Follow-up	Risk of bias	Inconsistency	Indirectness	Imprecision	Publication bias	Overall certainty of evidence	With control	With intervention	Relative effect (95% CI)	Risk with control	Risk difference with intervention
**Complete polyp regression rate- PDT VS Anti-VEGF**											
83 (2 RCTs)	serious [a,b]	not serious	not serious	not serious	none	⊕⊕⊕◯ MODERATE	29/44 (65.9%)	10/39 (25.6%)	**RR 0.46** (0.28 to 0.74)	660 per 1,000	356 fewer per 1,000 (475 fewer to 171 fewer)
**Complete polyp regression rate- PDT VS Early combination**											
78 (2 RCTs)	serious [a,b]	not serious	not serious	not serious	none	⊕⊕⊕◯ MODERATE	29/44 (65.9%)	20/34 (58.8%)	**RR 0.88** (0.58 to 1.33)	660 per 1,000	79 fewer per 1,000 (277 fewer to 218 more)
**Complete polyp regression rate- PDT VS Late combination**											
AIC	serious [a,b]	not serious	serious	not serious	none	⊕⊕◯◯ LOW			**RR 0.61** (0.36 to 1.03)		
**Complete polyp regression rate- Anti-VEGF VS Early combination**											
391 (3 RCTs)	serious [a,b]	not serious	not serious	not serious	none	⊕⊕⊕◯ MODERATE	62/190 (32.6%)	136/201 (67.7%))	**RR 1.91** (1.40 to 2.60)	327 per 1,000	298 more per 1,000 (131 more to 524 more)
**Complete polyp regression rate- Anti-VEGF VS Late combination**											
318 (1 RCT)	not serious	not serious	not serious	not serious	none	⊕⊕⊕⊕ High	61/157 (38.9%)	72/161 (44.8%)	**RR 1.33** (0.92 to 1.92)	389 per 1,000	129 more per 1,000 (31 fewer to 358 more)
**Complete polyp regression rate- Early combination VS Late combination**											
60 (1 RCTs)	serious [a,b]	not serious	not serious	not serious	none	⊕⊕⊕◯ MODERATE	18/29 (62.1%)	17/31 (54.8%)	**RR 0.70** (0.47 to 1.03)	621 per 1,000	186 fewer per 1,000 (329 fewer to 19 more)

PDT VS Anti-VEGF VS Early combination VS Late combination for PCV-adverse events

Certainty assessment							Summary of findings				
							Study event rates (%)			Anticipated absolute effects	
№ of participants (studies) Follow-up	Risk of bias	Inconsistency	Indirectness	Imprecision	Publication bias	Overall certainty of evidence	With control	With intervention	Relative effect (95% CI)	Risk with control	Risk difference with intervention
**Adverse events- PDT VS Anti-VEGF**											
83 (2 RCTs)	serious [a,b]	not serious	not serious	not serious	none	⊕⊕⊕◯ MODERATE	9/44 (20.5%)	8/39 (20.5%)	**RR 0.79** (0.41 to 1.53)	205 per 1,000	43 fewer per 1,000 (120 fewer to 109 more)
**Adverse events- PDT VS Early combination**											
78 (2 RCTs)	serious [a,b]	not serious	not serious	not serious	none	⊕⊕⊕◯ MODERATE	9/44 (20.5%)	7/34 (20.6%)	**RR 0.91** (0.47 to 1.48)	205 per 1,000	18 fewer per 1,000 (108 fewer to 99 more)
**Adverse events- PDT VS Late Combination**											
AIC	serious [a,b]	not serious	serious	not serious	none	⊕⊕◯◯ LOW			**RR 0.99** (0.75 to 1.29)		
**Adverse events- Anti-VEGF VS Early combination**											
402 (4 RCTs)	serious [a,b]	not serious	not serious	not serious	none	⊕⊕⊕◯ MODERATE	64/195 (32.8%)	80/207 (38.6%)	**RR 1.15** (0.89 to 1.48)	205 per 1,000	50 more per 1,000 (36 fewer to 108 more)
**Adverse events- Anti-VEGF VS Late combination**											
318 (1 RCTs)	not serious	not serious	not serious	serious [d]	none	⊕⊕⊕⊕ High	74/157 (47.1%)	64/161 (39.8%)	**RR 0.85** (0.66 to 1.09)	472 per 1,000	70 fewer per 1,000 (160 fewer to 43 more)
**Adverse events- Early combination VS Late combination**											
60 (1 RCTs)	serious [a,b]	not serious	not serious	not serious	none	⊕⊕⊕◯ MODERATE	1/31 (3.2%)	0/29 (0%)	**RR 0.74** (0.52 to 1.06)	368 per 1,000	8 fewer per 1,000 (15 fewer to 2 more)

Appendix 22

GRADE

PDT VS Anti-VEGF VS Early combination VS Late combination for PCV-ocular adverse events

Certainty assessment							Summary of findings				
							Study event rates (%)			Anticipated absolute effects	
№ of participants (studies) Follow-up	Risk of bias	Inconsistency	Indirectness	Imprecision	Publication bias	Overall certainty of evidence	With control	With intervention	Relative effect (95% CI)	Risk with control	Risk difference with intervention
**Ocular adverse events- PDT VS Anti-VEGF**											
83 (2 RCTs)	serious [a,b]	not serious	not serious	serious [d]	none	⊕⊕◯◯ LOW	8/44 (18.1%)	5/39 (12.8%)	**RR 0.83** (0.35 to 1.94)	182 per 1,000	30 fewer per 1,000 (118 fewer to 172 more)
**Ocular adverse events- PDT VS Early combination**											
83 (2 RCTs)	serious [a,b]	not serious	not serious	serious [d]	none	⊕⊕◯◯ LOW	8/44 (18.1%)	6/34 (17.6%)	**RR 0.93** (0.40 to 2.14)	181 per 1,000	12 fewer per 1,000 (109 fewer to 208 more)
**Ocular adverse events- PDT VS Late Combination**											
AIC	serious [a,b]	not serious	serious	serious [d]	none	⊕◯◯◯ LOW			**RR 0.78** (0.31 to 1.93)		
**Ocular adverse events- Anti-VEGF VS Early combination**											
402 (4 RCTs)	serious [a,b]	not serious	not serious	not serious	none	⊕⊕⊕◯ MODERATE	43/195 (22.1%)	52/207 (25.1%)	**RR 1.12** (0.79 to 1.58)	221 per 1,000	27 more per 1,000 (46 fewer to 129 more)
**Ocular adverse events- Anti-VEGF VS Late Combination**											
318 (1 RCTs))	serious [a,b]	not serious	not serious	not serious	none	⊕⊕⊕◯ MODERATE	49/157 (31.2%)	47/161 (29.2%)	**RR 0.94** (0.67 to 1.31)	181 per 1,000	18 fewer per 1,000 (103 fewer to 98 more)
**Ocular adverse events- Early combination VS Late Combination**											
AIC	serious [a,b]	not serious	serious	not serious	none	⊕⊕◯◯ LOW			**RR 0.84** (0.52 to 1.36)		

PDT VS Anti-VEGF VS Early combination VS Late combination for BCVA improvement rate

Certainty assessment							Summary of findings				
							Study event rates (%)			Anticipated absolute effects	
№ of participants (studies) Follow-up	Risk of bias	Inconsistency	Indirectness	Imprecision	Publication bias	Overall certainty of evidence	With control	With intervention	Relative effect (95% CI)	Risk with control	Risk difference with intervention
**BCVA improvement rate - PDT VS Anti-VEGF**											
176 (3 RCTs)	serious [a,b]	not serious	not serious	serious [d]	none	⊕⊕◯◯ LOW	19/91 (20.9%)	27/85 (31.8%)	**RR 1.26** (0.67 to 2.37)	209 per 1,000	55 more per 1,000 (68 fewer to 287 more)
**BCVA improvement rate - PDT VS Early combination**											
83 (2 RCTs)	serious [a,b]	not serious	not serious	serious [d]	none	⊕⊕◯◯ LOW	11/44 (25.0%)	9/34 (26.5%)	**RR 1.49** (0.71 to 3.13)	250 per 1,000	123 more per 1,000 (72 fewer to 533 more)
**BCVA improvement rate- PDT VS Late Combination**											
AIC	serious [a,b]	not serious	serious	serious [d]	none	⊕◯◯◯ LOW			**RR 1.61** (0.55 to 4.68)		
**BCVA improvement rate- Anti-VEGF VS Early combination**											
391 (3 RCTs)	serious [a,b]	not serious	not serious	serious [d]	none	⊕⊕◯◯ LOW	35/190 (18.4%)	50/201 (24.9%)	**RR 1.18** (0.64 to 2.16)	221 per 1,000	34 more per 1,000 (66 fewer to 215 more)
**BCVA improvement rate - Anti-VEGF VS Late Combination**											
AIC	serious [a,b]	not serious	not serious	serious [d]	none	⊕◯◯◯ LOW			**RR 1.27** (0.48 to 3.38)		
**BCVA improvement rate - Early combination VS Late Combination**											
60 (1 RCTs)	serious [a,b]	not serious	serious	serious [d]	none	⊕⊕◯◯ LOW	13/28 (44.8%)	15/31 (48.4%)	**RR 1.08** (0.50 to 2.33)		38 more per 1,000 (232 fewer to 619 more

Appendix 22

GRADE

Anti-VEGF VS Early combination VS Late combination for Anti-VEGF number

Certainty assessment							Summary of findings	
							Anticipated absolute effects	
№ of participants (studies) Follow-up	Risk of bias	Inconsistency	Indirectness	Imprecision	Publication bias	Overall certainty of evidence	Effect of control	Effect difference with intervention
**Anti-VEGF number - Anti-VEGF VS Early combination**								
788 (4 RCTs)	serious [a,b]	not serious [c]	not serious	not serious	none	⊕⊕⊕◯ MODERATE	The mean number ranged from **3.0 to 7.3**	MD **0.72 lower** (2.07 lower to 0.63 higher)
**Anti-VEGF number - Anti-VEGF VS Late Combination**								
318 (1 RCTs)	serious [a,b]	not serious [c]	not serious	not serious	none	⊕⊕⊕◯ MODERATE	The mean number ranged from 8.1 **to 8.1**	MD **1.44 lower** (3.61 lower to 0.75 lower)
**Anti-VEGF number - Early combination VS Late Combination**								
60 (1 RCTs)	serious [a,b]	not serious [c]	not serious	not serious	none	⊕⊕⊕◯ MODERATE	The mean number ranged from **6.8 to 6.8**	MD **0.72 lower** (2.02 lower to 1.48 higher)

CI: Confidence interval; **RR:** Risk ratio; **WMD:** Weighted mean difference

Explanations

a. Some trials have no information about the concealment of allocation sequence, or random method

b. The participants and people delivering the intervention in some trial are not masked, but it did not affect the outcome

c. High heterogeneity (I square> 50%).

d. wide confidence intervals

Appendix4. GRADE summary table
